# Optimal bailout strategies resulting from the drift controlled supercooled Stefan problem

**DOI:** 10.1007/s10479-023-05293-7

**Published:** 2023-04-29

**Authors:** Christa Cuchiero, Christoph Reisinger, Stefan Rigger

**Affiliations:** 1 Department of Statistics and Operations Research, Data Science @ Uni Vienna, Vienna University, Kolingasse 14-16, A-1090 Vienna, Austria; 2 Mathematical Institute and Oxford Man Institute of Quantitative Finance, University of Oxford, Andrew Wiles Building, Radcliffe Observatory Quarter, Oxford, OX2 6GG UK; 3 Department of Statistics and Operations Research, Vienna University, Kolingasse 14-16, A-1090 Vienna, Austria

**Keywords:** Systemic risk, Mean field control, Supercooled Stefan problem, Propagation of chaos, Bail-outs

## Abstract

We consider the problem faced by a central bank which bails out distressed financial institutions that pose systemic risk to the banking sector. In a structural default model with mutual obligations, the central agent seeks to inject a minimum amount of cash in order to limit defaults to a given proportion of entities. We prove that the value of the central agent’s control problem converges as the number of defaultable institutions goes to infinity, and that it satisfies a drift controlled version of the supercooled Stefan problem. We compute optimal strategies in feedback form by solving numerically a regularized version of the corresponding mean field control problem using a policy gradient method. Our simulations show that the central agent’s optimal strategy is to subsidise banks whose equity values lie in a non-trivial time-dependent region.

## Introduction

In this paper, we analyse a simple mathematical model for a central bank that optimally injects cash into a banking system with interbank lending in order to prevent systemic default events. By way of introduction, we first review known results on the dynamics without intervention and its relation to the supercooled Stefan problem. We then describe the optimisation problem faced by the central agent and discuss its setting within the literature on Mean Field Control (MFC) problems together with this paper’s contributions.

### Interbank lending and the supercooled Stefan problem

We study a market with *N* financial institutions and their equity value process $X=(X_t^i)$ for $t\in [0,T]$ with finite time horizon $T >0$ and $i=1,\ldots , N$. We interpret $X^i$ in the spirit of structural credit risk models as the value of assets minus liabilities. Hence, we consider an institution to be defaulted if its equity value hits 0. We refer the reader to Merton (1974) for the classical treatment as well as to Itkin and Lipton (2017) and the references therein for a discussion of such models in the present multivariate context with mutual obligations.

We consider specifically a stylised model of interbank lending where all firms are exchangeable, their equity values are driven by Brownian motion, and where the default of one firm leads to a uniform downward jump in the equity value of the surviving entities. The latter effect is the crucial mechanism for credit contagion in our model as it describes how the default of one firm affects the balance sheet of others. Here, we follow (Hambly et al., 2019; Nadtochiy & Shkolnikov, 2019; Lipton et al., 2019) to assume that the $X^i$ satisfy

1 $$\begin{aligned} X^i_t&= X^i_{0-} + B^i_t - \alpha \frac{1}{N} \sum _{i=1}^N \mathbb {1}_{\{\tau ^i \le t\}},\;\; \end{aligned}$$

where $\tau ^i = \inf \{t: X^i_t \le 0 \}$, $X^i_{0-}$ are non-negative i.i.d. random variables, $(B^i)_{1\le i\le N}$ is an *N*-dimensional standard Brownian motion, independent of $X_{0-}=(X^i_{0-})_{1\le i\le N}$, and $\alpha \ge 0$ is a given parameter measuring the interconnectedness in the banking system. The initial condition reflects the current state of the banking system. This might include minimal capital requirements prescribed by the regulator as conditions to enter the banking system, but we do not consider this question explicitly.

Even this highly stylised simple system produces complex behaviour for large pools of firms, including systemic events where cascades of defaults caused by interbank lending instantaneously wipe out significant proportions of the firm pool (see Hambly et al. (2019); Nadtochiy and Shkolnikov (2019); Delarue et al. (2022)).

One way of analysing this is to pass to the mean-field limit for $N \rightarrow \infty$. It is known (see, e.g., Delarue et al. (2015)) that the interaction (contagion) term in (1) converges (in an appropriate sense) to a deterministic function $\Lambda : [0,T] \rightarrow [0,\alpha ]$ as $N\rightarrow \infty$, i.e.,

$$\begin{aligned} \alpha \frac{1}{N} \sum _{i=1}^N \mathbb {1}_{\{\tau ^i \le t\}} \; \rightarrow \; \Lambda _t. \end{aligned}$$

Moreover, the $X^i$ are asymptotically independent with the same law as a process *X* which together with $\Lambda$ satisfies a probabilisitic version of the *supercooled Stefan problem*, namely

2 $$\begin{aligned} X_t = X_{0-} + B_t - \Lambda _t,\;\; t\ge 0, \end{aligned}$$

where $\Lambda$ is subject to the constraint

3 $$\begin{aligned} \Lambda _t = \alpha \mathbb {P}\Big (\inf _{0\le s\le t} X_s \le 0\Big ),\;\;t\ge 0. \end{aligned}$$

Here, *B* is a standard Brownian motion independent of the random variable $X_{0-}$, which has the same law as all $X^i_{0-}$. We refer to Delarue et al. (2022) for a discussion on how this probabilistic formulation relates to the classical PDE version of the supercooled Stefan problem.

From a large pool perspective (see Hambly et al. (2019); Nadtochiy and Shkolnikov (2019)), $X_t$ may be viewed as the equity value of a representative bank and $\tau = \inf \{t\ge 0: X_t \le 0\}$ as its default time, while $\Lambda _t$ describes the interaction with other institutions under the assumption of uniform lending and exchangeable dynamics. In particular, $\mathbb {P}(\inf _{0\le s\le t} X_s \le 0)$ can be interpreted as the fraction of defaulted banks at time *t* and consequently $\Lambda _t$ as the loss that the default of these entities has caused for the survivors.

It is known that solutions to (2), (3) are not unique in general (see Delarue et al. (2015); Cuchiero et al. (2023)), which explains the need to single out so-called *physical* solutions that are meaningful from an economic and physical perspective. Under appropriate conditions on $X_{0-}$, these physical solutions are characterised by open intervals with smooth $t \mapsto \Lambda _t$, separated by points at which this dependence may only be Hölder continuous or even exhibit a discontinuity, an event frequently referred to as *blow-up* (see Delarue et al. (2022)). If the mean of the initial values is close enough to zero relative to the interaction parameter $\alpha$, a jump necessarily happens (see Hambly et al. (2019)).

In case a discontinuity does occur at some $t\ge 0$, the following restriction on the jump size defines such a *physical solution*:

4 $$\begin{aligned} \Lambda _t-\Lambda _{t-}=\inf \Big \{x>0:\,\mathbb {P}\big (\tau \ge t,\,X_{t-}\in (0,x]\big )<\frac{x}{\alpha }\Big \},\;\;t\ge 0, \end{aligned}$$

with $\Lambda _{t-}:=\lim _{s\uparrow t} \Lambda _s$ and $X_{t-}:=\lim _{s\uparrow t} X_s$. By the results of Delarue et al. (2022), the above condition on the jumps of $\Lambda$ uniquely defines a solution to (2) and (3) under some restrictions on the initial condition $X_{0-}$. For future reference, we also introduce the concept of *minimal solutions*, which we know to be physical whenever the initial condition is integrable (see Cuchiero et al. (2023)). We call a solution $\underline{\Lambda }$ *minimal*, if for any other *X* that satisfies (2) with loss process $\Lambda$ given by (3), we have that

5 $$\begin{aligned} \underline{\Lambda }_t \le \Lambda _t, \quad t \ge 0. \end{aligned}$$

Note that by combining the results of Cuchiero et al. (2023) and Delarue et al. (2022) the minimal solution is the unique physical one, and thus economically meaningful one, if the initial condition satisfies the assumptions of Delarue et al. (2022).

### The central agent’s optimisation problem

The purpose of this paper is to analyse strategies that a central bank (*central agent*) can take to limit the number of defaults. They achieve this by controlling the rate of capital injected to distressed institutions. That is to say, rather than bailing out firms which are already defaulted, the central agent intervenes already ahead of the time their equity values become critical. This rate of capital^1^ received by bank *i* is determined by processes $\beta ^i$ and added to (1). In the finite dimensional situation, the $X^i$ then satisfy

6 $$\begin{aligned} X^i_t&= X^i_{0-} + \int _0^t \beta ^i_s \, ds + B^i_t - \alpha \frac{1}{N} \sum _{i=1}^N \mathbb {1}_{\{\tau ^i \le t\}}. \;\; \end{aligned}$$

A mathematically similar problem has been studied in Tang and Tsai (2018). There the question of finding an optimal drift in order to maximize the number of Brownian particles that stay above 0 is treated, however without the singular interaction term appearing in (6).

In anticipation of a propagation of chaos result (proved in Sect. 2), we therefore consider an extension of (2) and (3) with a drift process $\beta$, i.e.,

7 $$\begin{aligned} X_t&= X_{0-} + \int _0^t \beta _s \, ds + B_t - \Lambda _t,\;\; \end{aligned}$$

8 $$\begin{aligned} \Lambda _t&= \alpha \mathbb {P}\Big (\inf _{0\le s\le t} X_s \le 0\Big ).\;\; \end{aligned}$$

Throughout the paper we will consider a constraint $0 \le \beta _t \le b_{\text {max}}$, which amounts to the assumption that at any point in time the central agent has limited resources for the capital injections. We will specify further technical conditions on $\beta$ later, which allow us to show that indeed the finite system converges in a suitable sense to this McKean–Vlasov equation.

We now consider a central agent who injects capital into a representative bank at rate $\beta _t$ at time *t* in order to keep

$$\begin{aligned} L_{T-}(\beta ) = \mathbb {P}\Big (\inf _{0\le s< T} X_s \le 0\Big ) = \Lambda _{T-}(\beta )/\alpha , \end{aligned}$$

that is the number of defaults that occur before^2^ a given time *T*, below a specified threshold $\delta$, while minimising the expected total cost

$$\begin{aligned} C_T(\beta ) = \mathbb {E}\Big [ \int _0^T \beta _t \, dt \Big ]. \end{aligned}$$

We therefore consider the following constrained optimisation problem: For given $\delta$, the central agent solves

9 $$\begin{aligned} C_T(\beta ) \longrightarrow \min _{\beta } \qquad \text {subject to} \qquad L_{T-}(\beta ) \le \delta . \end{aligned}$$

Define now for $\gamma \in \mathbb {R}_+$ the Lagrange function $\mathcal {L}(\beta , \gamma ) = C_T(\beta )+ \gamma (L_{T-}(\beta ) - \delta )$ and use it to express the constrained optimization problem as an unconstrained one, namely $\min _{\beta } \max _{\gamma \in \mathbb {R}_+} \mathcal {L}(\beta , \gamma )$, which holds true since

$$\begin{aligned} \max _{\gamma \in \mathbb {R}_+} \mathcal {L}(\beta , \gamma )= {\left\{ \begin{array}{ll} C_T(\beta ) & \text {if } L_{T-} \le \delta \\ \infty & \text {else.} \end{array}\right. } \end{aligned}$$

Assuming the absence a duality gap^3^ (or equivalently the existence of a saddlepoint $(\beta ^\star , \gamma ^*)$ of $\mathcal {L}$, i.e. $\mathcal {L}(\beta ^\star , \gamma ) \le \mathcal {L}(\beta ^\star , \gamma ^*) \le \mathcal {L}(\beta , \gamma ^*)$ for all $\beta , \gamma$), then we can interchange the $\min$ and $\max$ and solve the dual problem $\max _{\gamma \in \mathbb {R}_+} \min _{\beta } \mathcal {L}(\beta , \gamma )$.

For these reasons we shall from now on consider the inner optimisation problem for fixed $\gamma >0$ (which can – due to the complementary slackness condition – only hold if the constraint is binding, i.e. $L_{T-}(\beta )=\delta$). If there is no duality gap, the optimal $\gamma$ for a prespecified threshold $\delta$ can in turn be determined by solving the outer optimisation problem, i.e. $\max _{\gamma \in \mathbb {R}_+} g(\gamma )$ where $g(\gamma )= \min _{\beta } \mathcal {L}(\beta , \gamma )$.

Writing ${\underline{X}}(\beta )$ for the solution process associated with the minimal solution $\underline{\Lambda }(\beta )$, analogously defined as in (5) but now for (7), we thus minimise the following objective function

10 $$\begin{aligned} \nonumber J(\beta )= &  \mathbb {E}\Big [ \int _0^T \beta _t \, dt \Big ] + \gamma \, \mathbb {P}\Big (\inf _{0\le s < T} {\underline{X}}_s(\beta ) \le 0\Big ) \\= &  \mathbb {E}\Big [ \int _0^T \beta _t \, dt + \gamma \, \mathbb {1}_{\{\widehat{{\underline{X}}}_{T-} = 0\}} \Big ], \end{aligned}$$

where $\widehat{{\underline{X}}}={\underline{X}}_t \mathbb {1}_{\{\tau > t\}}$ is the absorbed minimal solution process and $\tau$ the default time. Note that the only difference between $J(\beta )$ and $\mathcal {L}(\beta , \gamma )$ is the constant $-\gamma \delta$, which however does not play a role in the optimisation over $\beta$. By varying $\gamma$, we can therefore trace out pairs of costs and losses which are solutions to (9) for different $\delta$. The Lagrange multiplier $\gamma$ (as a function of $\delta$) can then be interpreted as shadow price of preventing further defaults. Indeed, as for usual constrained optimization problems, the optimal cost $C^{\star }_T$ seen as a function of the loss level $\delta$ satisfies under certain technical conditions

$$\begin{aligned} \partial _{\delta } C_T^{\star }(\delta )=\lim _{h \rightarrow 0}\frac{C^\star _T(\delta +h)-C^\star _T(\delta )}{h}= - \gamma (\delta ). \end{aligned}$$

As we show numerically in Sect. 3, the optimal loss $L_{T-}^\star$ as a function of $\gamma$ is monotone decreasing, so that for large enough $\gamma$ (and $b_{\max }$), the threshold $\delta$ becomes small enough to avoid systemic events.

Note that, by the arguments at the end of Sect. 1.1, using the minimal solution in the optimisation task is the only economically meaningful concept because non-physical solutions (with a potential higher probability of default) cannot be realistically justified, in particular when seen as limits of particle systems. We refer to Delarue et al. (2015, Section 3.1) for examples of such non-physical solutions.

Both from a theoretical and numerical perspective, we shall analyse the objective function (10) together with the dynamics (7), which is a non-standard *MFC problem with a singular interaction* through hitting the boundary. As we show in Sect. 2, in particular Theorem 2.8, optimisation of the McKean–Vlasov equation (7) yields the same result as first optimising in the *N*-particle system and then passing to the limit. In particular, by Theorem 2.10, optimizers of the McKean–Vlasov equation (7) are $\epsilon$-optimal for the *N*-particle system. This then justifies our numerical implementation described in Sect. 3 where we deal directly with the MFC problem.

### Relation to the literature

#### Theory of MFC problems and applications to systemic risk

Due to the big amount of literature on MFC problems we focus here on relatively recent works and mostly on MFC and not on the related concept of Mean Field Games (MFG) as introduced in Lasry and Lions (2007) and Huang et al. (2006). We refer to Carmona et al. (2013); Carmona and Delarue (2015); Carmona and Laurière (2021) for definitions of the MFC and MFG optima in general set-ups and discussions on the differences. As we here deal with a central agent our optimization problem corresponds to a Pareto optimum where all the agents cooperate to minimize the costs. Therefore MFC is the appropriate concept. Note that instead of MFC the terminology *McKean–Vlasov control* is often also used.

Similarly as for classical optimal control, dynamic programing principles have also been derived for MFC problems and can be found in Pham and Wei 2017; Djete et al. 2019. We also refer to Pham and Wei 2018; Achdou and Laurière 2015; Bensoussan et al. 2015; Laurière and Pironneau 2014, where in diffusion set-ups formulations using a Hamilton-Jacobi Bellman (HJB) equation on the space of probability measures for closed-loop controls (also called feedback controls) are deduced. In the recent work (Guo et al., 2020) this has been generalized to jump diffusion processes. For a dynamic programming principle for open-loop controls we refer to Bayraktar et al. (2018), and to Carmona and Delarue (2015), Acciaio et al. (2019) for a characterisation by a stochastic maximum principle.

We are here interested in feedback controls and would therefore need to solve the corresponding HJB equation (as e.g. in Pham and Wei (2018)), i.e. an infinite dimensional fully nonlinear partial differential equatios (PDE) of second order in the Wassertein space of probability measures. Solving such an equation is challenging, since it involves computing measure derivatives, which is numerically intractable. In our context the situation is even more intricate due to the singular interactions through the boundary. Indeed, even under the (usually not satisfied) assumption that $t \mapsto \Lambda _t$ is $C^1$, the problem is far beyond a standard MFC framework. In this case, $\Lambda$ in (7) can be replaced by $\int _0^{\cdot } \dot{\Lambda }_t dt$, thus a time derivative of the measure component, which makes the problem ‘non-Markovian’. Moreover, we deal with subprobability measures describing the marginal distributions of the absorbed process ${\widehat{X}}= X_t \mathbb {1}_{\{\tau > t\}}$ which governs the underlying dynamics. Note also that the total mass of these subprobability measures as well as ${\widehat{X}}$ itself can exhibit jumps if $\Lambda$ is discontinuous, and that these jumps emerge endogenously from the feedback mechanism.

This is in contrast to some other recent papers where jumps are exogenously given. For instance, the recent articles (Guo et al., 2020; Agram & Øksendal, 2021) consider the control of (conditional) McKean–Vlasov dynamics with jumps and associated HJB-PIDEs, while in Alasseur et al. (2021) a stochastic maximum principle is derived to analyse a mean-field game with random jump time penalty.

In the context of systemic risk and contagion via singular interactions through hitting times the paper (Nadtochiy & Shkolnikov, 2020) is especially relevant. There a game in which the banks determine their (inhomogeneous) connections strategically is analysed. It turns out that by a reduction of lending to critical institutions in equilibrium systemic events can be avoided. A model involving singular interaction through hitting the boundary is also considered in Elie et al. (2020). There, an optimization component is incorporated via a quadratic functional that allows the institutions to control parts of their dynamics in order to minimize their expected risk, which then leads to a MFG problem. The quadratic cost functional is inspired by the earlier work (Carmona et al., 2015), which also treats the mean-field game limit of a system of banks who control their borrowing from, and lending to, a central bank, and where the interaction comes from interbank lending. Let us finally mention the very recent article (Angiuli et al., 2022a) which applies reinforcement learning to a model that can be considered as an extension of Carmona et al. (2015) adding a cooperative game component within certain groups of banks.

In the wider context of interaction through boundary absorption, a few works on mean-field games have also appeared recently. In Campi and Fischer (2018), the players’ dynamics depends on the empirical measure representing players who have not exited a domain. This is extended to smooth dependence on boundary losses prior to the present time in Campi et al. (2019), and to the presence of common noise in Burzoni and Campi (2021). The economic motivation for these models are, among others, systemic risk and bank runs.

#### Numerics for MFC and MFG problems

Among the numerical methods proposed for MFC and MFG problems, we refer to Carmona and Laurière (2019) for a policy gradient-type method where feedback controls are approximated by neural networks and optimised for a given objective function; to Fouque and Zhang (2020) and again to Carmona and Laurière (2019) for a mean-field FBSDE method, generalising the deep BSDE method to mean-field dependence and in the former case to delayed effects; and to Achdou and Laurière (2020) for a survey of methods for the coupled PDE systems, mainly in the spirit of the seminal works (Achdou & Capuzzo-Dolcetta, 2010; Achdou & Laurière, 2016); see also a related semi-Lagrangian scheme in Carlini and Silva (2015) and a gradient method and penalisation approach in Pfeiffer (2016).

Beside these works on PDE systems, a lot of research has recently been conducted on how to apply (deep) reinforcement or Q-learning to solve MFC and MFG problems or combinations thereof (see e.g., Guo et al. (2019); Angiuli et al. (2021, 2022c, 2022b) and the references therein). We also refer to two recent survey articles (Hu & Lauriere, 2022; Laurière et al., 2022) on machine learning tools for optimal control and games. The first article focuses on methods that try to solve the problems by relying on exact computations of gradients exploiting the full knowledge of the model, while the second one presents learning tools that aim at solving MFC and MFG problems in a model-free fashion.

In our modeling and numerical approach we have full knowledge of the model, but what distinguishes it from the existing literature is the particular singular interaction through the boundary absorption. This means that all the discussed numerical schemes and methods need to be adapted to accommodate the current special situation. We opted for an adaptation of the policy gradient-type method considered in Reisinger et al. (2021a), since it shares the same computational complexity as the gradient-based algorithms in e.g. Archibald et al. (2020); Kerimkulov et al. (2021); Pfeiffer (2016), but enjoys an accelerated convergence rate and can handle general convex nonsmooth costs (including constraints), which allows to incorporate the current objective function. It exploits a forward-backward splitting approach and iteratively refines the approximate controls based on the gradient of the cost, evaluated through a coupled system of nonlocal linear PDEs. The precise algorithm is outlined in Sect. 3.

### Contributions and findings

As already mentioned, our model differs in a number of fundamental points from the existing literature: first, while (Carmona et al., 2015; Campi & Fischer, 2018; Campi et al., 2019; Nadtochiy & Shkolnikov, 2020; Elie et al., 2020; Burzoni & Campi, 2021) study mean-field game solutions and equilibria of *N*-player games, where each player maximises their own objective, we study the problem of a central planner who specifically seeks to control the number of defaults. Second, in contrast to Campi et al. (2019), where the coefficients of the players’ processes may depend on the loss process and to Burzoni and Campi (2021), which further includes a driver which is a smoothed version of *L* (hence modeling delayed effects of hitting the boundary), we consider the firm values driven by *L* directly, resulting in an instantaneous effect of defaults and the emergence of systemic events. Third, the techniques we use are also entirely different from those in these preceding works. Instead of relying on techniques for martingale problems used e.g. in Lacker (2017) to derive a limit theory for controlled McKean–Vlasov dynamics, we extend the method from Cuchiero et al. (2023) to show the convergence of the finite system to the mean-field limit.

Moreover, we provide a numerical solution adapting the approach of Reisinger et al. (2021a) to the current setting. This means first of all to express $\Lambda _t$ in the dynamics (7) and in the loss function explicitly in terms of the distribution of ${\widehat{X}}_t$, which can be (formally) achieved through $\partial _x p(t,0)$, i.e. the spatial derivative of its density at 0 (assuming it exists). To cast the absorbed process formally into a (more) standard McKean–Vlasov framework, instead of (7), we write the dynamics in a form where the drift and diffusion coefficients are multiplied with the Heaviside function (in the state). Both the computation of $\partial _x p(t,0)$ and the presence of the Heaviside function need some regularization, which is treated in Sect. 3.1. This regularized version then allows to apply the policy gradient descent method of Reisinger et al. (2021a) where we compute the gradient via a coupled forward-backward PDE system (instead of a particle method as in Reisinger et al. (2021a)). In particular, the forward problem is given by a smoothed version of the Stefan problem with a drift term determined by the feedback control, while the backward problem determines a decoupling field of the adjoint process.

From an economic point of view, our findings indicate a high sensitivity on the parameter $\gamma$ in (7). As shown in Fig. 7b, for a certain value of $\gamma$ and in a regime where $\alpha$ triggers jump discontinuities in the uncontrolled regime, the optimal control strategy switches from not avoiding a jump to avoiding a jump. Moreover, our numerical experiments suggest that it is not possible to vary the capital injection to control the *size* of the jump continuously, since the possible jump size is restricted by the physical jump constraint (4). Viewed differently, a large systemic event can happen if the central agent withdraws a small amount of capital from a scenario without jumps.

Summarizing, the main contributions of the present paper are as follows:

- We show convergence of the system with *N* agents to the mean-field limit (see Sect. 2), including well-posedness of the central agent’s optimisation problem, i.e. the existence of unique minimal solutions to the Stefan problem as given by (7)–(8) for any suitably regular control process $\beta$ and the existence of an optimal control which minimises (10).
- We propose a numerical scheme (see Sect. 3) based on policy gradient iteration, where the gradient is computed via coupled forward and backward PDEs satisfied by the density of a regularized version of the equity value process and a decoupling field corresponding to an adjoint process, respectively.
- We analyse by way of detailed numerical studies the structure of the central agent’s optimal strategy in different market environments, and the minimal losses that are attained under optimal strategies with varying cost (see also Sect. 3).

## Convergence to a mean-field limit

In this section, we show the existence of a minimising strategy for the central agent’s objective function in the mean-field limit, as well as convergence of the *N*-agent control problem.

### The model setup

We fix a measure $\nu \in \mathcal {P}([0,\infty ))$ and define a reference probability space to be a tuple ${\mathscr {S}} = (\Omega , \mathcal {F}, (\mathcal {F}_t)_{t\ge 0},\mathbb {P})$ such that ${\mathscr {S}}$ supports a Brownian motion that is adapted to $(\mathcal {F}_t)_{t\ge 0}$ and there is a $\mathcal {F}_0$-measurable random variable $X_{0-}$ with $\operatorname {law}(X_{0-}) = \nu$. Note that with this definition, $X_{0-}$ is independent of *B* by construction. We endow the space

11 $$\begin{aligned} S_T:=\{f \in L^2([0,\infty ))~|~ 0 \le f \le b_{\text {max}} ~\text {a.e.},~ f_{\vert _{(T,\infty )}} = 0\} \end{aligned}$$

with the topology of weak convergence in $L^2([0,\infty ))$. Since $S_T$ is bounded in the $L^2([0,\infty ))$-norm and weakly closed, $S_T$ is a compact Polish space. We then define the space of admissible controls

12 $$\begin{aligned} \mathcal {B}_T:= \{\beta \text { is } (\mathcal {F}_{t})_{t \ge 0}-\text {progressively measurable}~|~ \mathbb {P}(\beta \in S_T) = 1 \}. \end{aligned}$$

Note that the space of admissible controls $\mathcal {B}_T$ as well as the objective functional *J* as defined in (10) depend implicitly on the choice of stochastic basis ${\mathscr {S}}$. We will sometimes write $\mathcal {B}_T{({\mathscr {S}})}$ or $J^{({\mathscr {S}})}$ when we wish to emphasize this dependence. To be able to guarantee existence of optimizers and to make the optimization problem independent of the choice of stochastic basis ${\mathscr {S}}$, we will consider the relaxed optimization problem

13 $$\begin{aligned} V_\infty := \inf _{{\mathscr {S}}} \inf _{\beta \in \mathcal {B}_T ({\mathscr {S}})} J^{({\mathscr {S}})}(\beta ), \end{aligned}$$

as is standard in the stochastic optimal control literature (see e.g. Fleming and Soner (2006)). We say that $(X_{0-},B,\beta ,\underline{\Lambda })$ solve problem (7)–(8) on ${\mathscr {S}}$ if ${\mathscr {S}} = (\Omega , \mathcal {F}, (\mathcal {F}_t)_{t\ge 0},\mathbb {P})$ is a reference probability space with Brownian motion *B* and initial condition $X_{0-}$ such that $\beta \in \mathcal {B}_{T}^{({\mathscr {S}})}$ and (7)–(8) holds $\mathbb {P}$-almost surely.

Note that it is not clear a priori that the process *X* given in (7) is well-defined. Indeed, it is known that the McKean–Vlasov problem (2) and (3) may admit more than one solution, and it is not known that physical solutions exist for general $\beta \in \mathcal {B}_T$, although it is known for $\beta$ of the special form $b(t,X_t)$, where *b* is Lipschitz (see e.g. Delarue et al. (2015); Ledger and Søjmark (2021)). To pin down a meaningful solution concept, we therefore rely on the notion of *minimal solutions* as defined in (5). By the results of Cuchiero et al. (2023), we know that minimal solutions of the uncontrolled system are physical whenever the initial condition $X_{0-}$ is integrable.

Throughout the following sections $\mathcal {P}(E)$ always denotes the set of probability measures on a Polish space *E* which we endow with the Lévy-Prokhorov metric, i.e., convergence of probability measures is to be understood in the (probabilistic) weak sense. For function spaces etc. we apply rather standard notation and refer to Sect. A.1 for more details.

### Well-posedness of minimal solutions for general drift

We fix the reference probability space ${\mathscr {S}}$ and show that minimal solutions exist for any $\beta \in \mathcal {B}_T$. Define the operator $\Gamma$ for a càdlàg function $\ell$ and $\beta \in \mathcal {B}_T$ as

14 $$\begin{aligned} \left\{ \begin{aligned} X_t^{\ell }(\beta )&= X_{0-} + \int _{0}^{t}\beta _s \, ds + B_t - \alpha \ell _t, \\ \tau _{\beta }^{\ell }&= \inf \{t \ge 0: X_t^{\ell } (\beta )\le 0 \}, \\ \Gamma [\ell ,\beta ]_t&= \mathbb {P}({\tau _{\beta }^{\ell } \le t}). \end{aligned}\right. \end{aligned}$$

Note here that $(X^\ell (\beta ),\tau _{\beta }^\ell ,\ell )$ solves (7) if and only if $\ell$ is a fixed-point of $\Gamma [\cdot ,\beta ]$. We next introduce a function space that is mapped to itself by $\Gamma [\cdot ,\beta ]$: Set15where $\overline{\mathbb {R}}$ is the extended real line. Note that for $\ell \in M$, $\ell$ defines a cumulative distribution function of a probability measure on $[0,\infty ]$. Equipping *M* with the topology of weak convergence, i.e., we have that $\ell ^n \rightarrow \ell$ in *M* if and only if $\ell _{t}^n \rightarrow \ell _t$ for all $t \in [0,\infty ]$ that are continuity points of $\ell$, we obtain that *M* is a compact Polish space. As in the uncontrolled case, $\Gamma [\cdot ,\beta ]$ is continuous on *M*.

#### Theorem 2.1

For any $\beta \in \mathcal {B}_T$, the operator $\Gamma [\cdot ,\beta ]:M \rightarrow M$ is continuous. Furthermore, there is a (unique) minimal solution to (7), and it is given by

16 $$\begin{aligned} \underline{\Lambda }(\beta ) = \alpha \lim _{k\rightarrow \infty } \Gamma ^{(k)}[0,\beta ]. \end{aligned}$$

#### Proof

Using Lemma A.1, this follows from Theorem 2.3 in Cuchiero et al. (2022). $\square$

### Existence of an optimal control

A key step in proving existence of an optimizer is to show that sequences of solutions to (7)–(8) are compact in a certain sense and that their cluster points are solutions of (7)–(8). This is the content of the next theorem.

#### Theorem 2.2

Let $(X_{0-}^n,B^n,\beta ^n,\Lambda ^n)$ solve (7)–(8) on ${\mathscr {S}}^n$. Then, after passing to subsequences if necessary, there is a reference probability space ${\mathscr {S}}$ such that $(X_{0-},B,\beta ,\Lambda )$ solve (7)–(8) on ${\mathscr {S}}$ and it holds that $\operatorname {law}(\beta ^n) \rightarrow \operatorname {law}(\beta )$ in $\mathcal {P}(S_{T})$ and $\frac{1}{\alpha } \Lambda ^n \rightarrow \frac{1}{\alpha } \Lambda$ in *M*.

#### Proof

See Sect. A.2 in the Appendix. $\square$

#### Remark 2.3

Note that in Theorem 2.2, we do not assume that either the $\Lambda ^n$ or $\Lambda$ are minimal solutions. At this point, we do not know how to prove that $\Lambda$ is minimal if all $\Lambda ^n$ are minimal. Stability of the minimal solution is an open question (cf. [27, Conjecture 6.10]). For the proof of the subsequent theorem where we prove existence of an optimizer to (13), formulated with the minimal solution, this however does not matter.

Next, we prove that the infinite-dimensional problem (13) admits an optimizer.

#### Theorem 2.4

There is an optimizer of (13), i.e., there is a stochastic basis ${\mathscr {S}}^\star$ and $\beta ^\star \in \mathcal {B}_T ({\mathscr {S}}^{\star })$ such that

$$\begin{aligned} V_{\infty } = \inf _{{\mathscr {S}}} \inf _{\beta \in \mathcal {B}_T({\mathscr {S}})} J^{({\mathscr {S}})}(\beta ) = J^{({\mathscr {S}}^\star )}(\beta ^\star ). \end{aligned}$$

#### Proof

Let $(X_{0-}^n,B^n,\beta ^n,\Lambda ^n)$ be solutions to (7)–(8) on ${\mathscr {S}}^n$ such that ${J^{({\mathscr {S}}^n)}(\beta ^n) \le V_\infty + \frac{1}{n}}$. By Theorem 2.2, after passing to subsequences if necessary, there is a reference probability space ${\mathscr {S}}^\star$ such that $(X_{0-}^\star ,B^\star ,\beta ^\star ,\Lambda ^\star )$ solves (7)–(8) on ${\mathscr {S}}^\star$ and $\operatorname {law}(\beta ^n) \rightarrow \operatorname {law}(\beta ^\star )$ holds in $\mathcal {P}(S_T)$ as well as $\frac{1}{\alpha } \Lambda ^n \rightarrow \frac{1}{\alpha } \Lambda ^\star$ in *M*.

In the following, we simply write *J* instead of $J^{({\mathscr {S}}^\star )}$ etc. By construction, we have $J(\beta ^n) \le V_\infty + \frac{1}{n}$ and hence $\liminf _{n\rightarrow \infty } J(\beta ^n) \le V_\infty$. It is clear that $V_\infty \le \gamma < \infty$ by (13) and (10), as *J* attains a value less than or equal to $\gamma$ for $\beta =0$. We proceed to show that $J(\beta ^\star ) \le \liminf _{n\rightarrow \infty } J(\beta ^n)$.

Since the functional $b \mapsto \int _{0}^{T} b_s \;ds$ is continuous and bounded on $S_T$, it follows that

17 $$\begin{aligned} \lim _{n\rightarrow \infty } \mathbb {E}^n\left[ \int _{0}^{T} \beta _s^n\, ds \right] = \mathbb {E}^\star \left[ \int _{0}^{T}\beta _s^\star \,ds\right] . \end{aligned}$$

The Portmanteau theorem implies that

18 $$\begin{aligned} \liminf _{n\rightarrow \infty }\Lambda _{T-}^{n} \ge \Lambda _{T-}^{\star }, \end{aligned}$$

and since $\Lambda ^\star$ solves (7)–(8) with drift $\beta ^\star$ and $\underline{\Lambda }(\beta ^\star )$ is the minimal solution on ${\mathscr {S}}^\star$ with drift $\beta ^\star$, it follows that $\underline{\Lambda }_{T-}(\beta ^\star ) \le \Lambda _{T-}^\star$ which is equivalent to ${\underline{L}}_{T-}(\beta ^\star ) \le L_{T-}$, which concludes the proof. $\square$

### Properties of the controlled $N$-particle system

We describe the controlled $N$-particle system mentioned in the introduction in more detail. We consider a stochastic basis ${\mathscr {S}}_N = (\Omega _N, \mathcal {F}^N, (\mathcal {F}_t^N)_{t \ge 0}, \mathbb {P}_N)$ supporting certain exchangeable random variables, defined as follows.

#### Definition 2.5

Set $X^N:=(X^{1,N},\dots ,X^{N,N})$, where the $X^{i,N}$ are random variables taking values in some space *E*. We say that $X^N$ is *N*-exchangeable, if

$$\begin{aligned} \operatorname {law}(X^N)=\operatorname {law}((X^{\sigma (1),N},X^{\sigma (2),N},\dots ,X^{\sigma (N),N})), \end{aligned}$$

for any permutation $\sigma$ of $\{1,\dots ,N\}$. We say that $\beta ^N$ is ${\mathscr {S}}_N$-exchangeable if the vector $(X_{0-}^{i,N},B^{i,N},\beta ^{i,N})_{i=1}^{N}$ is *N*-exchangeable under $\mathbb {P}_N$.

The stochastic basis ${\mathscr {S}}_N$ is supposed to support an *N*-dimensional Brownian motion $B^N$ and an *N*-exchangeable, $\mathcal {F}_0^N$-measurable random vector $X_{0-}^N$. The particles in the system then satisfy the dynamics

19 $$\begin{aligned} X_{t}^{i,N} := X_{0-}^{i,N} + \int _{0}^{t} \beta _s^{i,N}\,ds + B_t^{i,N} - \Lambda _t^N, \end{aligned}$$

where $\beta ^N$ is ${\mathscr {S}}_N$-exchangeable, and $\Lambda _t^N = \frac{\alpha }{N} \sum _{i=1}^{N} \mathbb {1}_{\{\tau ^{i,N} \le t\}}$, where $\tau _{i,N}:= \inf \{t\ge 0: X_{t}^{i,N} \le 0\}$. In analogy to the infinite-dimensional case, we denote $L^N:= \frac{1}{\alpha } \Lambda ^N$. We then consider the set of admissible controls

20 $$\begin{aligned} \mathcal {B}_T^N:= \{\beta ^N \text { is }{\mathscr {S}}_N-\text { exchangeable, } (\mathcal {F}_{t}^N)_{t \ge 0}-\text {progressively measurable}~|~ \mathbb {P}(\beta ^{1,N} \in S_T) = 1 \}. \nonumber \\ \end{aligned}$$

The same examples as in the uncontrolled case show that solutions to (19) are not unique in general (cf. Section 3.1.1 in Delarue et al. (2015)). Therefore, in Delarue et al. (2015), physical solutions are introduced. Similarly to the infinite dimensional case we can also consider minimal solutions. We call a solution $\underline{\Lambda }^N$ to (19) minimal, if for every solution $\Lambda ^N$ to (19)

$$\begin{aligned} \underline{\Lambda }_t^N \le \Lambda _t^N, \quad t \ge 0, \end{aligned}$$

holds almost surely. The same argument as in [27, Lemma 3.3] shows that the notions of physical and minimal solution are equivalent in the controlled $N$-particle system. In analogy to the infinite-dimensional case, we introduce the operator

21 $$\begin{aligned} \left\{ \begin{aligned} X_{t}^{i,N}[{\textsf{L}},\beta ^N]&= X_{0-}^{i,N} + \int _{0}^{t} \beta _s^{i,N}\,ds + B_{t}^{i,N} -\alpha {\textsf{L}}_t \\ \tau _{i,N}[{\textsf{L}},\beta ^N]&= \inf \{t \ge 0: X_{t}^{i,N}[{\textsf{L}},\beta ^N] \le 0\} \\ \Gamma _N[{\textsf{L}},\beta ^N]_t&= \frac{1}{N}\sum _{i=1}^{N} \mathbb {1}_{\left\{ \tau _{i,N}[{\textsf{L}},\beta ^N]\le t\right\} }, \end{aligned}\right. \end{aligned}$$

where ${\textsf{L}}$ is some càdlàg process adapted to the filtration generated by $B^N$. We will often simply write $\Gamma _N[{\textsf{L}}]$ instead of $\Gamma _N[{\textsf{L}},\beta ^N]$. The statements are then meant to hold for arbitrary, fixed $\beta ^N$. An important property is that $\Gamma _N[\cdot ,\beta ^N]$ is monotone in the sense that

$$\begin{aligned} {\textsf{L}}^1_t \le {\textsf{L}}^2_t, \quad t \ge 0 \quad \implies \quad \Gamma _N[{\textsf{L}}^1,\beta ^N]_t \le \Gamma _N[{\textsf{L}}^2,\beta ^N]_t, \quad t \ge 0. \end{aligned}$$

We then readily see by straightforward induction arguments that

22 $$\begin{aligned} \alpha \Gamma _N^{(k)}[0] \le \Lambda ^N, \quad \Gamma _N^{(k)}[0] \le \Gamma _N^{(k+1)}[0], \quad k \in \mathbb {N}, \end{aligned}$$

holds almost surely, where $\Lambda ^N$ is any solution to the particle system and $\Gamma _N^{(k)}$ denotes the *k*-th iterate of $\Gamma _N$. A similar argument as for the system without drift in Cuchiero et al. (2023) shows that the iteration $(\Gamma _N^{(k)}[0])_{k\in \mathbb {N}}$ converges to the minimal solution after at most *N* iterations.

#### Lemma 2.6

For $N \in \mathbb {N}$, let $\Gamma _N$ be defined as in (21). Then $\underline{\Lambda }^N:=\alpha \Gamma ^{(N)}_N [0,\beta ^N]$ is the minimal solution to the particle system with drift $\beta ^N$ and the error bound

23 $$\begin{aligned} \Vert \alpha \Gamma ^{(k)}_N[0,\beta ^N] - \underline{\Lambda }^N\Vert _{\infty } \le \alpha \frac{(N-k)^+}{N} \end{aligned}$$

holds almost surely.

#### Proof

Analogous to the proof of Lemma 3.1 in Cuchiero et al. (2023). $\square$

Roughly speaking, the next result says that limit points (in distribution) of solutions to the controlled $N$-particle system converge (along subsequences) to solutions of the controlled McKean–Vlasov equation (7)–(8). By $D[-1, \infty )$ we denote here the space of cádlág paths on $[-1, \infty )$ equipped with the $M_1$-topology.

#### Theorem 2.7

For $N \in \mathbb {N}$, let $(X^N,\beta ^N,\Lambda ^N)$ be a solution to the particle system (19) on the stochastic basis ${\mathscr {S}}_N$ and define $\mu _N:= \frac{1}{N} \sum _{i=1}^{N} \delta _{X^{i,N}}$. Suppose that for some measure $\nu _{0-}\in \mathcal {P}(\mathbb {R})$ we have

$$\begin{aligned} \lim _{N\rightarrow \infty } \frac{1}{N}\sum _{i=1}^{N}\delta _{X_{0-}^{i,N}} = \nu _{0-}. \end{aligned}$$

Then there is a subsequence (again denoted by *N*) such that $\operatorname {law}(\mu _N) \rightarrow \operatorname {law}(\mu )$ in $\mathcal {P}(\mathcal {P}(D([-1,\infty ))))$, where $\mu$ coincides almost surely with the law of a solution process *X* to the McKean–Vlasov problem (7)–(8) satisfying $\operatorname {law}(X_{0-})=\nu _{0-}$.

#### Proof

See Sect. A.4. $$\square $$

The next theorem shows that when we optimize the policy in the particle system and then take the limit of the resulting optimal values, we obtain the same value that we find by optimizing the infinite-dimensional version of the problem.

#### Theorem 2.8

For $\kappa \in (0,1/2)$, define the value of a perturbed controlled $N$-particle system as

$$\begin{aligned} V_N&:= \inf _{{\mathscr {S}}_N} \inf _{\beta ^N \in \mathcal {B}({\mathscr {S}}_N)} J_N(\beta ^N), \quad J_N(\beta ^N) := \mathbb {E}^{N}\left[ \int _{0}^{T}\beta ^{1,N}_s\,ds + \gamma \underline{\tilde{L}} _{T-}^N(\beta ^N)\right] , \end{aligned}$$

where $\underline{\tilde{L}} ^N(\beta ^N):= \frac{1}{\alpha } \underline{\tilde{\Lambda }} ^N(\beta ^N)$ and $\underline{\tilde{\Lambda }} ^N(\beta ^N)$ is the minimal solution of the controlled $N$-particle system with drift $\beta ^N$ as introduced in Lemma 2.6 and perturbed initial condition ${\tilde{X}}_{0-}^{i,N} = X_{0-}^{i}+N^{-\kappa }$ for $\kappa \in (0,1/2)$ and all $i=1,\ldots ,N$. Then it holds that

24 $$\begin{aligned} \lim _{N\rightarrow \infty } V_N = V_{\infty }. \end{aligned}$$

#### Proof

Step 1: We show the inequality $\liminf _{N\rightarrow \infty } V_N \ge V_{\infty }$. To that end, choose ${\mathscr {S}}_N$ and $\beta ^N \in \mathcal {B}_T({\mathscr {S}}_N)$ such that

$$\begin{aligned} \mathbb {E}^{N}\left[ \int _{0}^{T}\beta ^{1,N}_s\,ds + \gamma \underline{\tilde{L}} _{T-}^{N} (\beta ^N)\right] \le V_N + \frac{1}{N}. \end{aligned}$$

Arguing as in the proof of Theorem 2.7, we see that $\xi _N = \frac{1}{N} \sum _{i=1}^{N} \delta _{(X_{0-}^{i}+N^{-\kappa }+B,\beta ^N,\underline{\tilde{L}} ^N (\beta ^N))}$ is tight, and by Theorem 2.7 converges to a limit $\xi$ that is supported on the set of solutions to the McKean–Vlasov problem (7)–(8). By Skorokhod representation, we may assume that this happens almost surely on some stochastic basis ${\mathscr {S}}$. Since the map $(w,b,\ell ) \mapsto \int _{0}^{T} b_s\,ds + \gamma \ell _{T-}$ is bounded and lower semicontinuous on $C([0,\infty )) \times S_T \times M$, Fatou’s Lemma and the Portmanteau theorem imply

$$\begin{aligned} \liminf _{N\rightarrow \infty }\mathbb {E}^{N}\left[ \int _{0}^{T}\beta ^{1,N}_s\,ds + \gamma \underline{\tilde{L}} _{T-}^N (\beta ^N)\right]&= \liminf _{N\rightarrow \infty } \mathbb {E}\left[ \int \left( \int _{0}^{T} b_s\,ds + \gamma \ell _{T-} \right) ~\textrm{d}\xi _N (w,b,\ell )\right] \\&\ge \mathbb {E}\left[ \liminf _{N\rightarrow \infty }\int \left( \int _{0}^{T} b_s\,ds + \gamma \ell _{T-} \right) ~\textrm{d}\xi _N (w,b,\ell )\right] \\&\ge \mathbb {E}\left[ \int \left( \int _{0}^{T} b_s\,ds + \gamma \ell _{T-} \right) ~\textrm{d}\xi (w,b,\ell )\right] . \end{aligned}$$

Defining ${\mathscr {S}}(\omega ) = (C([0,\infty ))\times S_T \times M, {\mathscr {B}}(C([0,\infty ))\times S_T \times M), \xi (\omega ))$, let $(w,b,\ell )$ denote the canoncial process on ${\mathscr {S}}$. By the arguments in the proof of Theorem 2.7 we have that *w* is Brownian motion under $\xi (\omega )$ with respect to the filtration generated by $(w,b,\ell )$, and we see that ${\mathscr {S}}(\omega )$ is an admissible reference space for almost every $\omega$. Since $\xi (\omega )$ corresponds to the law of a solution to the McKean–Vlasov problem (7)–(8), it follows that $\int \left( \int _{0}^{T} b_s\,ds + \gamma \ell _{T-} \right) ~\textrm{d}\xi (w,b,\ell ) \ge V_{\infty }$ almost surely. We have therefore obtained

25 $$\begin{aligned} \liminf _{N\rightarrow \infty } V_N \ge \liminf _{N\rightarrow \infty }\mathbb {E}^{N}\left[ \int _{0}^{T}\beta ^{1,N}_s\,ds +\gamma \underline{\tilde{L}} _{T-}^N(\beta ^N)\right] \ge V_{\infty }. \end{aligned}$$

Step 2: We show that $\limsup _{N\rightarrow \infty } V_N \le V_{\infty }$. Let ${\mathscr {S}}^\star$ be a probability space and $\beta ^{\star } \in \mathcal {B}({\mathscr {S}}^\star )$ be an optimizer attaining $V_{\infty }$, whose existence was shown in Theorem 2.4. Let ${\mathscr {S}}_N^\star$ be the product space obtained by taking *N* copies of ${\mathscr {S}}^\star$, and consider the (random) cost functional

26 $$\begin{aligned} c_N(b^N,\ell ) = \int _{0}^{T}b^{1,N}_s\,ds + \gamma \ell _{T-} + \gamma N^{\kappa } \Vert \Gamma _N[\ell ,b^N] - \ell \Vert _{\infty }, \quad b^N \in S_T^N, ~ \ell \in M, \end{aligned}$$

where $\Vert \cdot \Vert _{\infty }$ is the supremum norm on $[0,\infty )$. Let ${\textsf{M}}({\mathscr {S}})$ be the set of all $\mathcal {F}$-measurable random variables, defined on the stochastic basis ${\mathscr {S}}$, taking values in *M* and consider the problem

27 $$\begin{aligned} {\hat{V}}_N:= \inf _{\begin{array}{c} \beta ^N \in \mathcal {B}({\mathscr {S}}_N^\star ) \\ {\textsf{L}} \in {\textsf{M}}({\mathscr {S}}_N^\star ) \end{array}} \mathbb {E}_{\star }^{N}\left[ c_N(\beta ^N,{\textsf{L}})\right] . \end{aligned}$$

Letting $(\beta ^\star )^N$ be the vector obtained by taking *N* i.i.d. copies of $\beta ^\star$, and choosing ${\textsf{L}} \equiv {\underline{L}}(\beta ^\star )$, which we abbreviate in the following with ${\underline{L}}:= {\underline{L}}(\beta ^\star )$, we obtain

$$\begin{aligned} {\hat{V}}_N \le \mathbb {E}_{\star }^{N}\left[ \int _{0}^{T}\beta ^{\star }_s\,ds + \gamma {\underline{L}}_{T-} + \gamma N^{\kappa } \Vert \Gamma _N[{\underline{L}},(\beta ^\star )^N] - {\underline{L}}\Vert _{\infty }\right] . \end{aligned}$$

Noting that $\Gamma _N[{\underline{L}},(\beta ^\star )^N]$ is the empirical cumulative distribution function of the i.i.d. random variables $\tau _{\beta ^\star }^{i}:= \inf \{t \ge 0: X_{0-}^{i} + \int _{0}^{t} \beta _s^{\star ,i} \textrm{d}s + B_{t}^i - \underline{\Lambda }_t \le 0 \}$, and that $\mathbb {P}(\tau _{\beta ^\star }^i \le t) = {\underline{L}}_t(\beta ^\star )$, the same estimates as in Step 1 of the proof of Proposition 6.1 in Cuchiero et al. (2023) show that

$$\begin{aligned} \lim _{N\rightarrow \infty } \mathbb {E}\left[ N^{\kappa } \Vert \Gamma _N[{\underline{L}},(\beta ^\star )^N] - {\underline{L}}\Vert _{\infty }\right] = 0. \end{aligned}$$

We have therefore shown that

$$\begin{aligned} \limsup _{N\rightarrow \infty } {\hat{V}}_N \le \mathbb {E}_{\star }^{N}\left[ \int _{0}^{T}\beta ^{\star }_s\,ds + \gamma {\underline{L}}_{T-}(\beta ^\star )\right] = V_{\infty }. \end{aligned}$$

Now choose a sequence ${\hat{\beta ^N}} \in \mathcal {B}_T({\mathscr {S}}_N^\star ), {\textsf{L}}^N \in {\textsf{M}}({\mathscr {S}}_N^\star )$ such that $\mathbb {E}_{\star }^{N}[c({\hat{\beta ^N}},{\textsf{L}}^N)] \le {\hat{V}}_N + \frac{1}{N}$.

Define the sequence of events $A^N = \{\omega \in \Omega ^N :\Vert \Gamma _N[{\textsf{L}}^N,{\hat{\beta ^N]}} - {\textsf{L}}^N\Vert _{\infty } \le N^{-\kappa }\}$ and set $\hat{{\textsf{L}}}^N = {\textsf{L}}^N \mathbb {1}_{A^N} + {\underline{L}}^{N}({\hat{\beta }}^N) \mathbb {1}_{\Omega ^N {\setminus } A^N}$, where ${\underline{L}}^{N}({\hat{\beta }}^N)$ is the minimal solution on ${\mathscr {S}}^*_N$ with drift ${\hat{\beta }}^N$. With this choice, $\hat{{\textsf{L}}}^N$ is in ${\textsf{M}}({\mathscr {S}}^*_N)$ and satisfies

$$\begin{aligned} \Vert \Gamma _N[\hat{{\textsf{L}}}^N,{\hat{\beta ^N]}} - \hat{{\textsf{L}}}^N\Vert _{\infty } \le N^{-\kappa }, \quad \mathbb {E}_{\star }^{N}[c({\hat{\beta ^N}},\hat{{\textsf{L}}}^N)] \le \mathbb {E}_{\star }^{N}[c({\hat{\beta ^N}},{\textsf{L}}^N)] \le {\hat{V}}_N + \frac{1}{N}. \end{aligned}$$

Here we used that ${\underline{L}}^{N}({\hat{\beta }}^N) \le 1$ and $\Gamma _N[{\underline{L}}^{N}({\hat{\beta }}^N),\hat{\beta }^N] = {\underline{L}}^{N}({\hat{\beta }}^N)$. This implies

28 $$\begin{aligned} \hat{{\textsf{L}}}^N \ge \Gamma _N [\hat{{\textsf{L}}}^N,{\hat{\beta }}^N] - N^{-\kappa }. \end{aligned}$$

Since $\hat{{\textsf{L}}}^N \ge - N^{-\kappa }$, the monotonicity of $\Gamma _N$ implies that

29 $$\begin{aligned} \hat{{\textsf{L}}}^N \ge \Gamma _N[-N^{-\kappa },{\hat{\beta }}^N] - N^{-\kappa } = {\tilde{\Gamma }}_N [0,{\hat{\beta }}^N] - N^{-\kappa }, \end{aligned}$$

where ${\tilde{\Gamma }}_N$ is defined as in (21) with initial condition ${\tilde{X}}_{0-}^{i,N}:= X_{0-}^{i} + \alpha N^{-\kappa }$. Combining (29) with (28) and again using the monotonicity of $\Gamma _N$, we obtain

$$\begin{aligned} \hat{{\textsf{L}}}^N \ge \Gamma _N[{\tilde{\Gamma }}_N [0,{\hat{\beta }}^N] - N^{-\kappa }, {\hat{\beta }}^N] - N^{-\kappa } = {\tilde{\Gamma }}_N^{(2)}[0,{\hat{\beta }}^N] - N^{-\kappa }. \end{aligned}$$

A straightforward induction then shows that $\hat{{\textsf{L}}}^N \ge {\tilde{\Gamma }}_N^{(k)}[0,{\hat{\beta }}^N] - N^{-\kappa }$ for all $k \in \mathbb {N}$, and Lemma 2.6 then yields that we have

$$\begin{aligned} \hat{{\textsf{L}}}^N \ge \underline{\tilde{L}} ^N({\hat{\beta }}^N) - N^{-\kappa }, \end{aligned}$$

where $\underline{\tilde{L}} ^N({\hat{\beta }}^N)$ corresponds to the loss process associated to the particle system with initial condition ${\tilde{X}}_{0-}^{N}$. This yields

$$\begin{aligned} V_N - \gamma N^{-\kappa }&\le \mathbb {E}_{\star }^{N} \left[ \int _{0}^{T} \hat{\beta }_s^{1,N}\,ds + \gamma \underline{\tilde{L}} _{T-}^N(\hat{\beta }^N) \right] - \gamma N^{-\kappa } \le \mathbb {E}_{\star }^{N} \left[ \int _{0}^{T} \hat{\beta }_s^{1,N}\,ds + \gamma \hat{\textsf{L}}_{T-}^{N}\right] \\&\le \mathbb {E}_{\star }^{N} \left[ c(\hat{\beta }^N,\hat{\textsf{L}}^N)\right] \le \hat{V}_N + \frac{1}{N}. \end{aligned}$$

Since we have already shown that ${\limsup _{N\rightarrow \infty } {\hat{V}}_N \le V_{\infty }}$, this shows ${\limsup _{N\rightarrow \infty } V_N \le V_{\infty }}$, which concludes the proof. $\square$

#### Remark 2.9

We conjecture that the perturbation in the initial condition of the particle system in Theorem 2.8 is an artefact of our proof technique rather than a necessity.

#### Theorem 2.10

Let ${\mathscr {S}}^\star$ be a probability space and $\beta ^{\star } \in \mathcal {B}_T({\mathscr {S}}^\star )$ be an optimizer attaining $V_{\infty }$. Let ${\mathscr {S}}_N^\star$ be the product space obtained by taking *N* copies of ${\mathscr {S}}^\star$ and let $(\beta ^\star )^N$ be the vector obtained by taking *N* i.i.d. copies of $\beta ^\star$. Then, $(\beta ^\star )^N$ is $\epsilon$-optimal for the particle system, i.e., for every $\epsilon > 0$, it holds that

$$\begin{aligned} J_N((\beta ^\star )^N) \le V_N + \epsilon \end{aligned}$$

for *N* sufficiently large.

#### Proof

Let $\epsilon > 0$ be given. Recall the notation introduced in Step 2 of the proof of Theorem 2.8. Consider the problem

30 $$\begin{aligned} {\bar{V}}_N:= \inf _{{\textsf{L}} \in {\textsf{M}}({\mathscr {S}}_N^\star )} \mathbb {E}_{\star }^{N}\left[ c_N((\beta ^\star )^N,{\textsf{L}})\right] . \end{aligned}$$

Proceeding as in Step 2 of the proof of Theorem 2.8, it follows that $\limsup _{N\rightarrow \infty } {\bar{V}}_N \le V_{\infty }$. By Theorem 2.8, we have $\lim _{N \rightarrow \infty } V_N = V_{\infty }$, and therefore $\bar{V}_N \le V_N + \epsilon / 3$ for *N* large enough. Arguing as in Step 2 of the proof of Theorem 2.8, we can find ${\textsf{L}}^N \in {\textsf{M}}({\mathscr {S}}_N^\star )$ such that $\mathbb {E}[c_N((\beta ^\star )^N, {\textsf{L}}^N)] \le \bar{V}_N + \epsilon /3$ and ${{\textsf{L}}}^N \ge \underline{\tilde{L}} ^N((\beta ^\star )^N) - N^{-\kappa }$ holds. Choosing *N* large enough such that $\gamma N^{-\kappa } < \epsilon /3$, we obtain

$$\begin{aligned} J_N((\beta ^\star )^N)\le \mathbb {E}_{\star }^{N} \left[ \int _{0}^{T} \beta ^\star _s\,ds + \gamma \hat{{\textsf{L}}}_{T-}^{N}\right] + \gamma N^{-\kappa } \le \bar{V}_N + 2\epsilon /3 \le V_N +\epsilon . \end{aligned}$$

$$\square $$

## Numerical solution of the MFC problem

In this section, we present a numerical scheme for the central agent’s mean-field control problem. We directly compute the optimal feedback control by a policy gradient method (PGM; see Sect. 3.2 and  3.3) applied to a regularised version of the dynamics and the objective function (see Sects. 3.1). The gradient is approximated by finite difference schemes for the density of the forward process and a decoupling field for an adjoint process (see Sect. 3.4). This will allow us to conduct parameter studies of the optimal strategies as well as the resulting losses and costs in Sect. 3.5.

Recall from (10) and above the process ${\underline{X}}(\beta )$ corresponding to the minimal solution $\underline{\Lambda }(\beta )$, and write the objective function as

31 $$\begin{aligned} J(\beta ) = \mathbb {E}\Big [ \int _0^T (\beta _t + \gamma {\dot{L}}_t) \, dt \Big ], \end{aligned}$$

where $L_t = \mathbb {P}\Big (\inf _{0\le s < T} {\underline{X}}_s(\beta ) \le 0\Big )$, and derivatives of *L* are defined in a distributional sense if necessary.

In the case of regular solutions, the absorbed process associated with (7), $\widehat{{\underline{X}}}={\underline{X}}_t \mathbb {1}_{\{\tau > t\}}$, for $\tau$ the hitting time of 0, has a sub-probability density *p* supported on $(0,\infty )$ and an atomic mass at 0. Similarly as in Delarue et al. (2022, Theorem 1.1), it satisfies the forward Kolmogorov equation

32 $$\begin{aligned} \begin{aligned}&\partial _t p + \partial _x (\beta p)=\frac{1}{2}\partial _{xx}p+{\dot{\Lambda }}_t\partial _x p,\;\;x\ge 0,\;\;t\in \mathbb {T}, \\&p(0,x)=f(x),\;\; x\ge 0\quad \text {and}\quad p(t,0)=0,\;\;t\in \mathbb {T}, \end{aligned} \end{aligned}$$

where

33 $$\begin{aligned} \begin{aligned} \Lambda _t = \alpha \Big (1 - \int _0^\infty p(t,x)\, dx \Big ), \qquad t\in \mathbb {T}, \end{aligned} \end{aligned}$$

and where $\mathbb {T}$ denotes the set of all $t\in [0,T]$ where $t\rightarrow L_t$ is differentiable. If $t\notin \mathbb {T}$, in particular in the event of a blow-up at *t*, we have the following jump condition for the solution of (32), $p(t-,x) = p(t,x-\Lambda _t + \Lambda _{t-})$.

Assuming again regular enough solutions where we can take the derivative with respect to time of the equation ${L_t = 1- \int _{0}^{\infty } p(t,x)~dx}$, we find that

$$\begin{aligned} {\dot{L}}_t = -\int _{0}^{\infty }\partial _{t}p~{d}x = -\int _{0}^{\infty } \frac{1}{2} \partial _{xx}p~{d}x -\int _{0}^{\infty } \alpha {\dot{\Lambda }}\partial _{x}p~{d}x = \frac{1}{2} \partial _{x} p(t,0). \end{aligned}$$

Moreover, we can rewrite the controlled dynamics of $\widehat{{\underline{X}}}$ for $t<\tau$ as

34 $$\begin{aligned} dX_t&= (\beta _t - \alpha {\dot{L}}_t) \, dt + dB_t. \end{aligned}$$

Note that the current control problem lies outside the standard MFC context due to the following three main aspects: (i) the interaction through the boundary leads to a time derivative of the measure component, which makes the problem as written in (34) (without replacing $\dot{L}_t$ by $\partial _x p(t,0)/2$) ‘non-Markovian’; (ii) the drift coefficient is non-Lipschitz in the measure component; (iii) the dynamics are defined by an absorbed process, which moreover has an irregular drift coefficient (as $t\rightarrow L_t$ can be discontinuous in time). We will address these points by a regularisation in the next section, which will subsequently allow us to apply a policy gradient method, which is inspired by Reisinger et al. (2021b).

### Regularisation

Denote by $\nu _t$ the law of $\widehat{{\underline{X}}}_t$ corresponding to *p*(*t*, *x*)*dx* in the regular case where a density exists. For (small) $h>0$, we approximate $\frac{1}{2}p_x(t,0)$ in terms of the measure $\nu _t$ by

$$\begin{aligned} {\dot{L}}^h_t = \frac{1}{2} \int _{-\infty }^\infty (- \partial \phi ^h)(x) \, \nu _t(dx)= \frac{1}{2}\langle - \partial \phi ^h, \nu _t \rangle , \end{aligned}$$

where $\phi ^h(x)$ is a smooth approximation of the Dirac $\delta$ distribution with support in [0, *h*] and where the bracket notation is used to denote the integral.

We then define a smooth function $\Phi ^h: \mathbb {R} \rightarrow \mathbb {R}$ such that $\Phi ^h(x)=0$ for $x\le - \kappa h$ and $\Phi ^h(x)=1$ for $x\ge 0$, for some $\kappa >0$, and consider the dynamics

35 $$\begin{aligned} dX_t&= a^h(X_t,\beta _t,\nu _t) \, dt + \sigma ^h(X_t) \, dB_t, \end{aligned}$$

where

$$\begin{aligned} a^h(x,b,\nu ) = \Phi ^h(x) \left( b +\frac{\alpha }{2} \langle \partial \phi ^h, \nu \rangle \right) , \qquad \sigma ^h(x) = \Phi ^h(x). \end{aligned}$$

For completeness, we give the specific $\phi ^h$ and $\Phi ^h$ used in our computations in Appendix B.1. There, we also show the graphs of $\phi ^h$ and its first two derivatives for the value $h=10^{-3}$, which is frequently used in our tests below.

Under the dynamics (35), the process does not get absorbed at 0, but once it crosses 0 from above its diffusion and drift coefficients decay rapidly so that with high probability it remains in the interval $[-\kappa h,0]$ (see Fig. 4 for an illustration of the density of such a process).

The reason why we consider these modified dynamics is to cast the absorbed process $\widehat{{\underline{X}}}_t$ into a standard McKean–Vlasov framework. The objective function can also be rewritten and becomes

36 $$\begin{aligned} J(\beta )&= \mathbb {E}\Big [ \int _0^T f^h(\beta _t,\nu _t) \, dt \Big ], \qquad f^h(b,\nu ) = b + \frac{\gamma }{2} \langle - \partial \phi ^h, \nu \rangle . \end{aligned}$$

A crucial point here is that both the coefficients in the dynamics and the objective can be written in terms of $\beta$ and $\nu$ alone, i.e. without any time (or spatial) derivatives of the measure flow $(\nu _t)$. Note also that $(a^h, \sigma ^h, f^h)$ satisfy the differentiability assumptions made Acciaio et al. (2019, Section 3), which we shall need for the Fréchet differentiability of the function *F* defined in (40) below.

Once we have the optimal control in feedback form $\beta ^\star _t = \beta ^\star (t,x)$ for $\beta ^\star : [0,T] \times [0,\infty ) \rightarrow [0,b_{\max }]$, and the associated density *p* of $\widehat{{\underline{X}}}$, we can compute the optimal loss and cost pair as

37 $$\begin{aligned} L^\star _T= &  \frac{1}{2} \int _0^T \int _{-\infty }^\infty (- \partial \phi ^h)(x) \ p(t,x) \, dx dt, \end{aligned}$$

38 $$\begin{aligned} C^\star _T= &  \int _0^T \int _{-\infty }^\infty \beta ^\star (t,x) \ p(t,x) \, dx dt. \end{aligned}$$

### Policy gradients

We follow here in spirit the approach of Reisinger et al. (2021b). We consider first a slightly more general form of the MFC problem, written as a nonsmooth optimization problem over the Hilbert space $\mathcal {H}^2({\mathbb R})$ of ${\mathbb R}$-valued square integrable, progressively measurable processes,

39 $$\begin{aligned} \inf _{\beta \in \mathcal {H}^{2}(\mathbb {R})} ( F(\beta )+G(\beta ) ), \end{aligned}$$

with the functionals $F:\mathcal {H}^2(\mathbb {R})\rightarrow {\mathbb R}$ and $G:\mathcal {H}^2(\mathbb {R})\rightarrow {\mathbb R}\cup \{\infty \}$ defined as follows: for all $\beta \in \mathcal {H}^2({\mathbb R})$,

40 $$\begin{aligned} F(\beta ){:}{=}\mathbb {E}\bigg [ \int _0^T f^h(\beta _t,\nu _t) \, \textrm{d}t \bigg ], \quad G(\beta ){:}{=}\mathbb {E}\bigg [ \int _0^T g(\beta _t) \, \textrm{d}t\bigg ], \end{aligned}$$

where $f^h$ is defined in (36).

The splitting of the objective function into *F* and *G* allows for a separate treatment of the smooth component $f^h$ and a non-smooth component *g*. We will use *g* to incorporate the constraints on $\beta$, specifically, $g(x) = 0$ for $x \in [0,b_{\max }]$ and $\infty$ outside. It is clear that $G:\mathcal {H}^2(\mathbb {R})\rightarrow {\mathbb R}\cup \{\infty \}$ is convex due to the convexity of *g*.

Assuming that $\nu _t$ lies in the Wasserstein space of probability measures on $\mathbb {R}$ with finite second moment, denoted by $\mathcal {P}_2(\mathbb {R})$, we introduce the Hamiltonian $H: \mathbb {R}\times \mathbb {R}\times \mathcal {P}_2(\mathbb {R})\times \mathbb {R}\times \mathbb {R}\rightarrow \mathbb {R}$ by

41 $$\begin{aligned} H(x,b,\nu ,y,z){:}{=}a^h(x,b,\nu ) y + \sigma ^h(x) z + f^h(b,\nu ), \end{aligned}$$

with

42 $$\begin{aligned} \partial _x H(x,b,\nu ,y,z)= \partial \Phi ^h(x) \left( b + \frac{\alpha }{2} \langle \partial \phi ^h, \nu \rangle \right) y + \partial \Phi ^h(x) z. \end{aligned}$$

Moreover, by Acciaio et al. (2019, Lemma 3.1), $F:\mathcal {H}^2(\mathbb {R})\rightarrow {\mathbb R}$ is Fréchet differentiable and its derivative $\nabla F:\mathcal {H}^2(\mathbb {R})\rightarrow \mathcal {H}^2(\mathbb {R})$ satisfies for all $\beta \in \mathcal {H}^2({\mathbb R})$,

43 $$\begin{aligned} {(\nabla F)(\beta )_t}= (\partial _{b} H)(X^{\beta }_t,\beta _t, \nu _t, Y^{\beta }_t, Z^{\beta }_t) + \tilde{\mathbb {E}}[ (\partial _{\nu } H)({\tilde{X}}^{\beta }_t, {\tilde{\beta }}_t, \nu _t, {\tilde{Y}}^{\beta }_t, {\tilde{Z}}^{\beta }_t)(X^{\beta }_t)], \end{aligned}$$

$\textrm{d}t\otimes \textrm{d}{\mathbb P}$-a.e. Here, $X^\beta$ is the state process controlled by $\beta$, satisfying (35), and $(Y^{\beta }, Z^{\beta })$ are square integrable adapted adjoint processes such that for all $t\in [0,T]$,

44 $$\begin{aligned} \begin{aligned} \textrm{d}Y^{\beta }_t&= (-\partial _x H(X^{\beta }_t,\beta _t,\nu _t,Y_t^{\beta }, Z_t^{\beta }) -{\tilde{\mathbb {E}}}[(\partial _{\nu } H)({\tilde{X}}^{\beta }_t, {\tilde{\beta }}_t,\nu _t,{\tilde{Y}}^{\beta }_t, {\tilde{Z}}^{\beta }_t)(X^{\beta }_t)] )\,\textrm{d}t +Z^{\beta }_t\, d W_t, \\ Y^{\beta }_T&= 0. \end{aligned} \end{aligned}$$

Above and hereafter, we use the tilde notation to denote an independent copy of a random variable as in Acciaio et al. (2019).

We now consider controls in feedback form, namely $\beta _t = \beta (t,X_t)$, which determine $X^\beta _t$ as solution of

45 $$\begin{aligned} \textrm{d} X_t= a^h(X_t, \beta (t,X_t),\nu _t) \, dt + \sigma ^h(X_t) \, dW_t. \end{aligned}$$

Then a sufficiently smooth decoupling field *u* such that $Y_t= u(t,X_t)$ and $Z_t=\sigma ^h(X_t) \partial _x u(t,X_t)$ satisfies

46 $$\begin{aligned} \begin{aligned}&\partial _t u + \frac{1}{2} \sigma ^h(x)^2 \partial _x^2 u + a^h(x, \beta (t,x),\nu ) \partial _x u = -\partial _x H(x,\beta (t,x),\nu ,u,\sigma ^h(x) \partial _x u)\\&\qquad \qquad \qquad \qquad \quad - {\tilde{\mathbb {E}}}[(\partial _{\nu } H)({\tilde{X}}_t, \beta (t,{\tilde{X}}_t),\nu _t,u(t,{\tilde{X}}_t), \sigma ^h({\tilde{X}}_t) \partial _x u(t,{\tilde{X}}_t))(x)], \end{aligned} \end{aligned}$$

with terminal condition $u(T,\cdot ) = 0$.

#### Computation of gradient by decoupling fields

In our application, we can express the right-hand side of (46) more explicitly. For the Hamiltonian (41) with $a^h$ and $f^h$ defined by (35) and (36), respectively, we have, by Carmona et al. (2018, Section 5.2.2, Example 1)

$$\begin{aligned} (\partial _{\nu } H)({\tilde{X}}_t, {\tilde{\beta }}_t,\nu _t,{\tilde{Y}}_t, {\tilde{Z}}_t)(X_t)= &  (\partial _{\nu }a^h)({\tilde{X}}_t, {\tilde{\beta }}_t,\nu _t)(X_t) \, {\tilde{Y}}_t + (\partial _{\nu } f^h)({\tilde{\beta }}_t,\nu _t)(X_t) \\= &  \frac{\alpha }{2} \Phi ^h({\widetilde{X}}_t) \partial ^2 \phi ^h(X_t) {\tilde{Y}}_t - frac{\gamma }{2} \partial ^2 \phi ^h(X_t), \\ - {\tilde{\mathbb {E}}}[(\partial _{\nu } H)({\tilde{X}}_t, {\tilde{\beta }}_t,\nu _t,{\tilde{Y}}_t, {\tilde{Z}}_t)(X_t)]= &  \frac{1}{2} (\gamma - \alpha {\tilde{\mathbb {E}}}[\Phi ^h({\widetilde{X}}_t) {\tilde{Y}}_t]) \, \partial ^2 \phi ^h(X_t) \\= &  \frac{1}{2} \left( \gamma - \alpha \langle \Phi ^h u(t, \cdot ), \nu _t \rangle \right) \partial ^2 \phi ^h(X_t). \end{aligned}$$

Consequently, for the decoupling field *u*, with $z=\sigma ^h \partial _x u= \Phi ^h\partial _x u$,

$$\begin{aligned}&\partial _t u + \frac{1}{2} \sigma ^h(x)^2 \partial _x^2 u + a^h(x,\beta (t,x),\nu _t) \partial _x u = - \partial \Phi ^h(x) (\beta (t,x) +\frac{\alpha }{2} \langle \partial \phi ^h, \nu _t \rangle ) u \\&\quad - \partial \Phi ^h(x) \Phi ^h(x)\partial _x u + \frac{1}{2} \left( \gamma - \alpha \langle \Phi ^h u(t, \cdot ), \nu _t \rangle \right) \partial ^2 \phi ^h(x), \end{aligned}$$

which can be re-written as

$$\begin{aligned} &  \partial _t u +\frac{1}{2} \, \partial _x\! \left( \Phi ^h(x)^2 \partial _x u \right) + (\beta (t,x) +\frac{\alpha }{2} \langle \partial \phi ^h, \nu _t \rangle ) \, \partial _x\!\left( \Phi ^h(x) u \right) \\  &  = \frac{1}{2} \left( \gamma \!-\! \alpha \langle \Phi ^h u(t, \cdot ), \nu _t \rangle \right) \partial ^2 \phi ^h(x). \end{aligned}$$

As $(\partial _{b} H)(x,b, \nu , y,z) = \Phi ^h(x) y + 1$, we obtain

47 $$\begin{aligned} (\nabla F)(\beta )(t,x)= \Phi ^h(x) u(t,x) + 1 - \frac{1}{2} \left( \gamma - \alpha \langle \Phi ^h u(t, \cdot ), \nu _t \rangle \right) \partial ^2 \phi ^h(x), \end{aligned}$$

where we will assume that $\nu _t$ has a density $p(t,\cdot )$ which satisfies

48 $$\begin{aligned} \partial _t p + \partial _x \left( a^h(x, \beta (t,x), \nu _t) p \right) = \frac{1}{2} \partial _x^2 (\sigma ^h(x)^2 p). \end{aligned}$$

### A proximal policy gradient method (PGM)

We now compute a sequence of approximations to the optimal control in feedback form, namely $\beta ^m_t = \beta ^m(t,X^m_t)$. Following (Reisinger et al., 2021b), we will carry out proximal gradient steps with $\beta ^0$ given, e.g. zero, and thereafter, for step size $\tau >0$,

49 $$\begin{aligned} \begin{aligned} \beta ^{m+1}(t,x)&= \text {prox}_{\tau g} \left( \beta ^m(t,x)-\tau (\nabla F)(\beta ^{m})(t,x) \right) , \end{aligned} \end{aligned}$$

where $\mathop {\textrm{prox}}\limits _{\tau g}:{\mathbb R}^k\rightarrow {\mathbb R}^k$ is the proximal map of $\tau g:{\mathbb R}\rightarrow {\mathbb R}\cup \{\infty \}$ such that

$$\begin{aligned} \text {prox}_{\tau g}(b)=\arg \min _{z\in {\mathbb R}}\left( \frac{1}{2}|z-b|^2+\tau g(z)\right) , \quad a\in {\mathbb R},\tau >0. \end{aligned}$$

For the considered *g*, an indicator function, $\mathop {\textrm{prox}}\limits$ is simply the projection onto $[0,b_{\max }]$, i.e. $\text {prox}_{\tau g}(b)= \min (\max (b,0),b_{\max })$.

Then a sufficiently smooth decoupling field $u^m$ such that $Y^m_t= u^m(t,X_t^m)$ satisfies

50 $$\begin{aligned} \begin{aligned}&\partial _t u^m + \frac{1}{2} \, \partial _x\! \left( \Phi ^h(x)^2 \partial _x u^m \right) + (\beta ^m(t,x) +\frac{\alpha }{2} \langle \partial \phi ^h, \nu ^m_t \rangle ) \, \partial _x\!\left( \Phi ^h(x) u^m \right) \\ &= \frac{1}{2} \left( \gamma \!-\! \alpha \langle \Phi ^h u^m(t, \cdot ), \nu ^m_t \rangle \right) \partial ^2 \phi ^h(x), \end{aligned} \end{aligned}$$

where $\nu ^m(dx) = p^m \, dx$ for the density $p^m$ that satisfies

51 $$\begin{aligned} \partial _t p^m + \partial _x \left( a^h(x,\beta ^{m}(t,x), \nu ^m) p^m \right) = \frac{1}{2} \partial _x^2 \Phi ^h(x)^2 p^m, \end{aligned}$$

and where

$$\begin{aligned} a^h(x,\beta ^{m}(t,x), \nu ^m) =\Phi ^h(x) \left( \beta ^{m}(t,x) + \frac{\alpha }{2} \int _{-\infty }^\infty \partial \phi ^h(x) \, p^m(t,x) \, dx \right) . \end{aligned}$$

Finally,

52 $$\begin{aligned} {(\nabla F)(\beta ^m)}(t,x)= \Phi ^h(x) u^m(t,x) + 1 - \frac{1}{2} \left( \gamma - \alpha \langle \Phi ^h u^m(t, \cdot ), \nu ^m_t \rangle \right) \partial ^2 \phi ^h(x). \end{aligned}$$

### Numerical implementation

We pick regularisation parameters $h,\kappa >0$ for $\phi ^h$ and $\Phi ^h$ defined as above. Then in the *m*-th iteration, we first solve numerically (51) for $p^m$ and then (46) for $u^m$, where $\nu ^m$ is the measure with density $p^m$. We use a semi-implicit finite difference scheme on a non-uniform mesh, as detailed below.

We define a numerical approximation on a time mesh $t_i = i \Delta t$, $i\in \mathbb {I}= \{0,1,\ldots , N\}$, $\Delta t = T/N$ for a positive integer *N*.

We also define a non-uniform spatial mesh $(x_j)_{j\in \mathbb {J}}$ with $\mathbb {J}= \{0,1,\ldots ,J\}$, for $x_0 = x_{\min } <0$, $x_J = x_{\max }>0$.

In the following, we drop the iteration index *m* and use instead superscript *i* to denote the timestep of any function defined on the space-time mesh and subscript *j* its spatial index, in particular, for the numerical PDE solutions, $p_j^i\approx p(t_i,x_j)$, $u_j^i\approx u(t_i,x_j)$. We assume a feedback control $b_j^i = \beta (t_i,x_j)$ is defined on this mesh.

Starting with the forward equation (51), for each $x_j$ and $t_i$, we approximate the drift coefficient *a* by

53 $$\begin{aligned} a_j^i = \Phi ^h(x_j) \left( b_j^i - \alpha L^i \right) \quad \text { for } \quad L^i = - \frac{1}{2} \sum _{k=0}^{J-1} \partial \phi ^h(x_k) \, p_k^{i-1} \, (x_{k+1}-x_k), \end{aligned}$$

and set $s_j^i = \Phi ^h(x_j)^2$. Then define a finite difference scheme by $p_j^0 = f(x_j)$, and for $i>0$,

$$\begin{aligned} \frac{p^i_j-p^{i-1}_j}{\Delta t} + \frac{\max (a_{j}^i,0) p_{j}^i - \max (a_{j-1}^i,0) p_{j-1}^i}{x_{j}-x_{j-1}} + \frac{\min (a_{j+1}^i,0) p_{j+1}^i - \min (a_{j}^i,0) p_{j}^i}{x_{j+1}-x_{j}} = &  \\ \frac{1}{x_{j+1}-x_{j-1}} \left( \frac{s^i_{j+1} p^i_{j+1} - s^i_{j} p^i_{j}}{x_{j+1}-x_{j}} - \frac{s^i_{j} p^i_{j} - s^i_{j-1} p^i_{j-1}}{x_{j}-x_{j-1}} \right) , \qquad 0< j < J, &  \\ p^i_j = 0, \qquad \text {else}. &  \end{aligned}$$

This is an upwind scheme for the first order terms, taking the appearance of *p* in *a* explicit, but otherwise implicit. The form of the scheme is chosen to be consistent with (51) for non-uniform meshes, in particular where the mesh size is piecewise constant.

For the adjoint equation (47), with $p_j^i$ now given in addition to $b_j^i$, we first define the right-hand side,

54 $$\begin{aligned} r_j^i = \frac{1}{2} \left( \gamma - \alpha \sum _{k=0}^{J-1} \partial \Phi ^h(x_k) \, u^{i+1}_k p_k^{i} \, (x_{k+1}-x_k) \right) \partial ^2 \phi ^h(x_j), \end{aligned}$$

and then, with $u_j^N = 0$, we define for $i<N$

$$\begin{aligned} &  \frac{u^{i+1}_j-u^{i}_j}{\Delta t} + \min (a_{j}^i,0) \frac{u_{j}^i - u_{j-1}^i}{x_{j}-x_{j-1}} + \max (a_{j}^i,0) \frac{u_{j+1}^i - u_{j}^i}{x_{j+1}-x_{j}} \\ &  \qquad -\frac{1}{x_{j+1}-x_{j-1}} \left( s^i_{j+1/2} \frac{u^i_{j+1} - u^i_{j}}{x_{j+1}-x_{j}}- s^i_{j-1/2} \frac{u^i_{j} - u^i_{j-1}}{x_{j}-x_{j-1}} \right) = r_j^i, \qquad 0< j < J, \\ &  u^i_j = 0, \qquad \text {else}. \end{aligned}$$

This allows us to compute the gradient on the same mesh, from (52),

$$\begin{aligned} G_j^i = \Phi ^h(x_j) u_j^i + 1 - r_j^i, \end{aligned}$$

and perform updates $b_j^i \leftarrow \min (\max (b_j^i - \tau G_j^i,0),b_{\max }).$

Finally, (37) is approximated by *L* in (53) and (38) by

55 $$\begin{aligned} \frac{T}{N} \sum _{i=1}^N \sum _{j_0}^{J-1} b_j^i \ p_j^i \ (x_{j+1}-x_j), \qquad j_0 = \min \{j: x_j>0\}. \end{aligned}$$

**Fig. 1:**
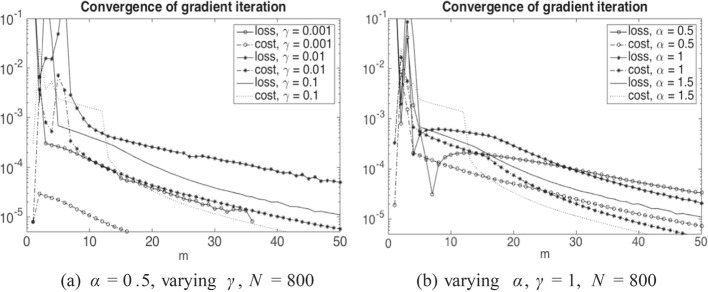
Convergence of *C* and *L* in the PGM for varying $\gamma$ and $\alpha$. Shown are $|L^{(m+1)} - L^{(m)}|$ and $|C^{(m+1)} - C^{(m)}|$

Let us remark that we do not have a convergence proof for this numerical scheme and it also seems out of reach due to the delicate interplay between the discretization and regularization parameters visible from Table 1. Nevertheless, for fixed *N* and *h* we can empirically show convergence of the gradient iterations (see Fig. 1), which then allows us to compute approximate optimal policies. In this sense our numerical tests indicate at least qualitatively how the optimal policies look like. Note that a rigorous convergence proof of a similar policy gradient iteration method in the non-mean field regime has recently been provided in Reisinger et al. (2022).

#### Set-up and model parameters

In the rest of the paper, we give illustrations of the model’s suggested strategies and resulting loss behaviour in different market scenarios, influenced by the interaction parameter $\alpha$, the risk aversion $\gamma$, the initial state *f*, and maximum cash injection rate $b_{\max }$.

In all examples, we choose a gamma initial density,

56 $$\begin{aligned} f(x) = 1/\Gamma (k) \theta ^{-k} x^{k-1} \textrm{e}^{-x/\theta }, \qquad x\ge 0. \end{aligned}$$

The parameters of the initial distribution could be calibrated to CDS spreads if they are traded (see Bujok and Reisinger (2012)). The default parameters we use are $k=2$, $\theta =1/3$, chosen to give a range of different behaviours by varying the other parameters. In this case, *f* is differentiable with $f(0+)=0$. This choice implies that there are smooth solutions for a short enough time interval (see Hambly et al. (2019); Delarue et al. (2022)). It also implies (see Hambly et al. (2019, Theorem 1.1)) that a blow-up (of the unregularised system) is guaranteed to happen at some time for $\alpha > 2 \mathbb {E}[X_{0-}] = 2 k\theta = 4/3$. Conversely, it is known (see Ledger et al. (2020, Theorem 2.2 and the comment below it)) that the condition $\alpha \Vert f\Vert _{\infty } < 1$ leads to the so-called weak feedback regime, where continuity of solutions always holds true.

A simple estimation of meaningful $\alpha$ from typical asset volatilities, recovery rates, and mutual lending as proportion of overall debt is found in Lipton et al. (2019), suggesting possible values from 0.3 to possibly higher than 5. We shall conduct tests for $\alpha \in \{0.5, 1, 1.5\}$. With $\Vert f\Vert _{\infty } \approx 1.1$, it is clear that a jump cannot occur for $\alpha =0.5$, but is guaranteed for $\alpha = 1.5$ as then $2 \mathbb {E}[X_{0-}]<\alpha$. The terminal time is chosen as $T=0.02$. We find empirically that the uncontrolled system does not jump in this interval for $\alpha =1$ (although it may jump eventually), and does jump halfway through the interval for $\alpha =1.5$. We have intentionally chosen an initial distribution where blow-ups can happen at such relatively short time scales to illustrate the different effects. In our regularised version of the problem, this manifests in a smooth transition to high values of losses, around 60%, over a short period of time. We fix $b_{\max }=10$ at first, and investigate the effect of larger values later on.

In the following, when not stated otherwise, we choose $\kappa = 1/10$ in the construction of $\Phi ^h$ (see 3.1 and Appendix B.1), which was found a reasonable choice in our experiments. As default, solutions are computed with $N=800$ timesteps and a non-uniform mesh on $[x_{\min },x_{\max }] = [-2,6]$ which is constructed as described below.

#### Mesh convergence

We first analyse the convergence of the finite difference approximations for fixed control. In particular, we first choose $\beta = 0$. The interaction parameter is $\alpha =0.5$.

The mesh is chosen uniformly in the intervals $[x_{\min },-0.02]$, $[-0.02,0.05]$, $[0.05,x_{\max }]$, such that approximately 5% of the points lie in the first interval, 45% in the second, and 50% in the third, and the total number $N_x$ of spatial mesh points is approximately $N \cdot (x_{\max }-x_{\min })/(8 T)$. This has the effect that the average mesh size is roughly eight times the time step size, which turns out a reasonable ratio in our numerical tests.

It is of crucial importance to have enough mesh points in the intervals $[-\kappa h,0]$ and [0, *h*] to approximate the smoothed Heaviside function and the smoothed delta distribution with its first two derivatives. A strong local mesh refinement as above allows this while keeping the total computational complexity feasible. Notice for our choice above the local mesh size around zero is almost 100 times smaller than for larger *x*.

In Table 1, we report for a varying number of time-steps *N* (and proportionally chosen $N_x$) and smoothing parameter *h* the computed loss (columns 5–10, rows 3–8). Let $L_N^h$ be the loss computed with *N* time steps and parameter *h*. Then from the table we conjecture convergence of $L_N^h$ as $N\rightarrow \infty$ for fixed *h*, but divergence as $h\rightarrow 0$ for fixed *N*.

**Table 1:** Mesh convergence, losses, $\alpha =1.5$ and $\gamma =0.1$

			$$10^3 \cdot h $$	1	$$2^{-1}$$	$$2^{-2} $$	$$2^{-3}$$	$$2^{-4} $$	$$2^{-5} $$	CPU (s)
$\frac{N}{10^2}$	$N_x/10^3$	$10^3 \cdot \theta _N$	$\rho _N$							
1	3.75	1.956	-2.42	0.5643	0.6430	0.7137	0.8324	4.7756	0	0.44
2	7.5	-0.806	1.31	0.5663	0.6260	0.6693	0.7268	0.8384	5.1210	1.2
4	15	-0.614	1.82	0.5655	0.6164	0.6481	0.6778	0.8223	0.0336	4.2
8	30	-0.337	1.94	0.5649	0.6118	0.6376	0.6589	0.6884	0.6096	17
16	60	-0.173	–	0.5645	0.6096	0.6327	0.6486	0.6647	0.6893	83
32	120	–	–	0.5643	0.6085	0.6304	0.6440	0.6548	0.6680	427
			$10^2 \cdot \vartheta _h$	4.414	2.192	1.354	1.084	1.322	–	
			$\varrho _h$	2.01	1.61	1.24	0.81	–	–	

To investigate this more quantitatively, we report in the third and fourth columns $\theta _N = L_{2 N}^{h}-L_{N}^{h}$ and $\rho _N = \theta _N/\theta _{2 N}$, where $h = 10^{-3}$. The fact that, for fixed *h*, the increments $\theta _N$ for successive mesh refinements decrease inversely proportional to *N* is consistent with first order convergence in 1/*N* and $1/N_x$. Conversely, we fix $N=3200$ and examine $\vartheta _h = L_{N}^{h/2}-L_{N}^{h}$ and $\varrho _h = \vartheta _{h}/\vartheta _{h/2}$ in the last two rows. The behaviour indicates a decrease of first order in *h* as long as $1/N_x$ is small compared to *h*, but divergence thereafter. Finally, the approximate computational times, reported in the last column, are approximately linear in $N N_x$ and independent of *h*.^4^

A similar behaviour is observed for the approximation of the cost and for different parameters, as shown in Appendix B.2.

#### Convergence of policy gradient iteration (PGM)

Next, we analyse the convergence of the policy gradient iteration. Here and thereafter, we will use a modification whereby (49) is evaluated for $x > h$, while $\beta ^{m+1} = b_{\max }$ for $x\le h$. As the occupation time of [0, *h*] is small, the effect of this choice has a negligible effect on the expected cost in all cases. We found that this modified iteration converged faster and more reliably in our numerical tests.

We monitor in each iteration the loss at time *T* computed as in (53), and the expected cost, computed as in (55). For $L^{(m)}$ and $C^{(m)}$ the terminal loss and total expected cost at the *m*-th iteration, respectively, we plot in Fig. 1 the steps $|L^{(m+1)} - L^{(m)}|$ and $|C^{(m+1)} - C^{(m)}|$. In these tests, the iteration terminates if either both of these quantities are smaller than $10^{-5}$ or 50 iterations are reached.

The left-hand plot in Fig. 1a shows the convergence for different values of $\gamma \in \{10^{-3}, 10^{-2}, 10^{-1}\}$. The intermediate value of $\gamma$ has the largest absolute error, while the smallest $\gamma$ leads to the smallest one. In the latter case, the cost is very small due to the very small penalty of losses. The asymptotic rate of convergence appears similar for all parameters considered.

In Fig. 1b, we analyse the effect of $\alpha$ on the convergence. The error is largest for the smallest of $\alpha \in \{0.5, 1, 1.5\}$, while the error is smallest for $\alpha =1.5$, which is the case where a jump occurs in an uncontrolled setting and losses are the largest.

Further parameter studies are given in the Appendix, where Fig. 10a establishes robustness of the convergence under mesh refinement and Fig. 10b illustrates the effect of the step size.

In most situations, the number of iterations required for reasonable accuracy, i.e. a relative error below around $10^{-3}$ was between 10 and 30, so that for the chosen discretisation (with $N=800$ timesteps and mesh as chosen above) the computing time to solve the MFC problem was between 3 and 10 min on the laptop as specified earlier.

### Computational analysis of central agent’s strategy

We now move to an analysis of the optimal strategies, and the achievable pairs of costs and losses under the optimal and other strategies.

#### Analysis of the optimal strategy

The policy gradient method produces directly an approximation to the optimal feedback control $\beta ^\star$. We found that an initialisation of the iteration with a function of the form $\beta ^0(t,x)=b_{\max }$ for $0<x<c$ and 0 elsewhere, for some $c>0$ large enough so that the support of $\beta ^0$ covers the support of $\beta ^\star$, produces more regular controls for small iteration numbers than a zero initialisation. The following plots were produced with $c=0.2$ and a tolerance $10^{-5}$ in the loss and cost (compare Fig. 1a).

**Fig. 2:**
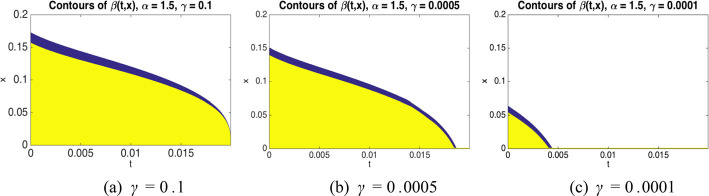
Contour plots of $(t,x) \rightarrow \beta ^\star (t,x)$ for $\alpha =1.5$ and different $\gamma$. The white region is $\{\beta ^\star \le 0.05 \ b_{\max }\}$, the (yellow) shaded region $\{\beta ^\star \ge 0.95 \ b_{\max }\}$, the dark (blue) zone the transition

We depict in Fig. 2 contours of the optimal feedback control $\beta ^\star (t,x)$ for different $\gamma$. As expected from the form of the Hamilton-Jacobi-Bellmann equation, the control is close to a ‘bang-bang’ structure, i.e. a piecewise constant function where the control always takes one of the two extreme values,i.e. either 0 or $b_{\text {max}}$. The two regions are separated by a narrow strip where the control transitions continuously. We conjecture this to be an effect of the numerical procedure, which is designed for Lipschitz continuous feedback controls.

The (yellow) shaded region closest to $x=0$ is where $\beta ^\star (t,x) \ge 0.95 \ b_{\max }$, i.e. the central agent subsidises firms closest to default at or close to the maximum rate. The white region furthest from $x=0$ is where $\beta ^\star (t,x) \le 0.05 \ b_{\max }$, i.e. the central agent does not subsidise firms with high reserves.

For larger $\gamma$, here exemplified by $\gamma = 0.1$ in Fig. 22, the contribution of the loss to the objective is large enough for the central agent to act for all *t*, for values in *x* up to a decreasing curve in *t*. Close to the chosen end point, the effect of the control on the overall losses becomes negligible and does not justify the associated cost. In a sense this behaviour is an artifact of the finite observation interval.

**Fig. 3:**
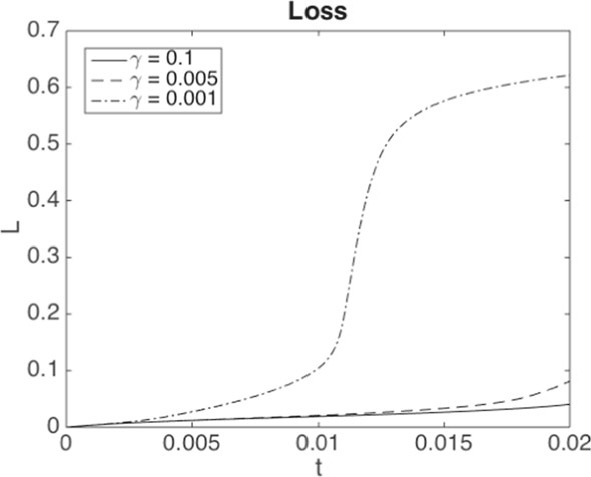
Loss for $$\gamma \in \{0.1, 0.005, 0.001\}$$

For smaller $\gamma$, in Figs. 2b and 2c, the agent only acts (for sufficiently small states) up to a certain point in time and then does nothing. Combining this with the plots of the resulting loss curves in Fig. 3, a possible interpretation is that the agent seeks to delay the onset of the strongly contagious phase until it is no longer viable to do with a certain cost budget, depending on $\gamma$. In particular, as visible from Fig. 3, under the current optimization criterion the jump is not avoided for $\gamma =0.001$. We discuss at the end of the next subsection other strategies for avoiding jumps – for the current optimisation criterion such a strategy is however not necessarily optimal.

**Fig. 4:**
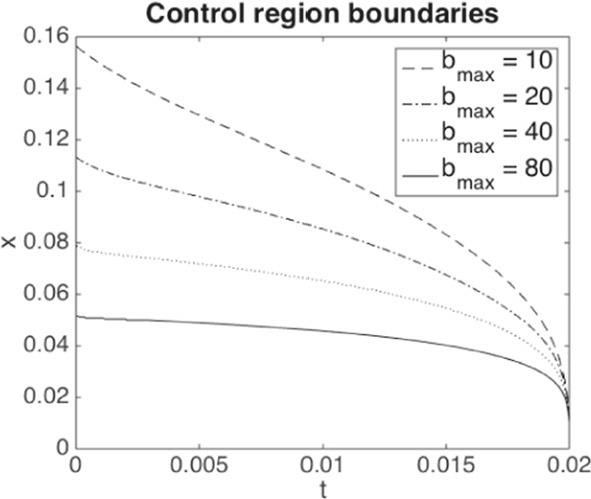
Control regions for different $b_{\max }$

Next, we analyse the impact the upper bound of $b_{\max }$ on the control strategy. We show only the $0.99\ b_{\max }$ level set for clarity in Fig. 4, for different $b_{\max }$. The region under this curve indicates where the agent controls at (or close to) the maximum rate. The region shrinks as $b_{\max }$ increases, meaning that the agent is able to control the banks’ equity process more effectively whenever it gets close to zero.

**Fig. 5:**
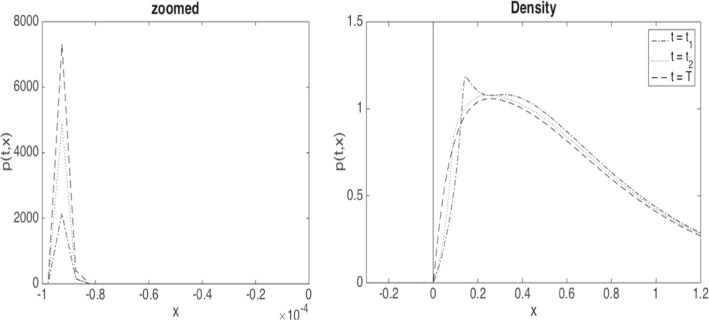
Parameters $\alpha =1.5$, $\gamma = 0.1$. Left: Density $p(t,\cdot )$ for small negative *x*. Right: Density $p(t,\cdot )$ in macroscopic range

Lastly in this section, we analyse the behaviour of the PDE solutions *p* and *u* at different times and on different scales in *x* around 0. Figure 5 shows in the left panel the accumulation of probability mass in the interval $[-c h,0]$ due to the smooth truncation of the SDE coefficients, approximating the absorption at $x=0$. As can be deduced from the right plot in Fig. 5, the area under the density for positive *x* is thus reduced, but only by a small amount in the current parameter setting (see Fig. 3 for the corresponding loss function with $\gamma =0.1$).

**Fig. 6:**
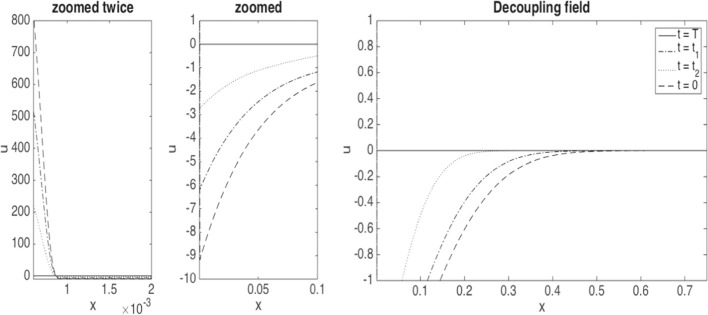
Parameters $\alpha =1.5$, $\gamma = 0.0005$. Left and middle: Decoupling field $u(t,\cdot )$ for different *t* and two ranges of (small) *x*. Right: Decoupling field $u(t,\cdot )$ for different *t* and marcroscopic range

In Fig. 6 we illustrate the behaviour of *u* on different scales in *x*. The left-most plot shows the range $[0,2\,h]$, where *u* attains large positive values; in the middle plot, over [0, 0.1], *u* has moderate negative values; the right-most plot is truncated below by $-1$, this being the threshold which determines where the control is active. This can be seen from (52) in conjunction with (49): for $x>h$, the gradient is $u+1$, so for a converged control we have $\beta = b_{\max }$ where $u+1<0$ and $\beta = 0$ where $u+1>0$. From this the bang-bang structure of the control becomes also clear.

#### Analysis of optimal cost-loss pairs

Finally, we examine the pairs of costs and losses that are obtained under the optimal policy and other heuristic strategies.

**Fig. 7:**
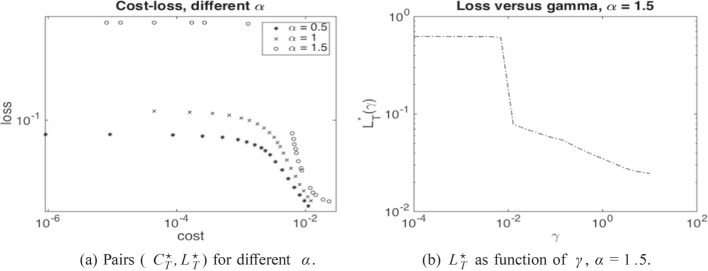
Cost $C^\star _T$ and loss $L^\star _T$ in the optimal regime for logarithmically spaced $\gamma \in [0.0001, 0.1]$ and different $\alpha$ in **a** and the dependence of the losses on $\gamma$ in **b**

In Fig. 7a, we vary $\gamma$ to trace out the curve $(C^\star _T(\gamma ), L^\star _T(\gamma ))$, where $C^\star _T(\gamma )$ and $L^\star _T(\gamma )$ are the costs and losses given by (37) and (38) for the chosen $\gamma$. For a given cost, the graph gives the loss achievable under the optimal strategy. To achieve a smaller loss, a higher cost is generally incurred.

We focus first on the data for $\alpha =0.5$ and $\alpha =1$. In these cases, the uncontrolled system exhibits no jumps and cash injection simply reduces the losses. For small $\gamma$, minimising the cost is the priority and the losses approach those of the uncontrolled system. For growing $\gamma$, it becomes favourable to increase the cash injection and a significant reduction of losses can be achieved. This levels off for large $\gamma$ as the cap $b_{\max }$ on the cash injection rate limits the overall effect of bail-outs.

For strong interaction, here exemplified by $\alpha =1.5$, we observe a discontinuity, which is further analysed in Fig. 7b. For $\gamma$ around 0.01, the optimal strategy switches from not preventing a jump to preventing a jump. This is manifested in Fig. 7b by an downward discontinuity in the number of losses. The optimal value of the central agent’s control problem is also discontinuous in $\gamma$ at this point. In other words, it is not possible to vary the capital injection to control the *size* of the jump continuously. Rather, the possible jump size is restricted by the constraint (4) on physical solutions. Conversely, withdrawal of a small amount of cash by the central agent from a scenario with low losses can trigger a large systemic event.

Note that Fig. 7b also allows to deduce the relation between $\gamma$ and the threshold $\delta$ by looking for $\gamma$ such that $\gamma \in \operatorname {argmax}_{\gamma \in \mathbb {R}_+} C^\star _T(\gamma ) + \gamma (L^\star _T(\gamma ) - \delta )$, as explained in the introduction. Indeed, this corresponds to solving the outer optimization problem $\max _{\gamma \in \mathbb {R}_+} g(\gamma )$ where $g(\gamma )= \min _{\beta } \mathcal {L}(\beta , \gamma )$ with $\mathcal {L}(\beta , \gamma )$ denoting the Lagrange function. Under the assumption of no duality gap and a unique optimizer $\gamma$, $\gamma$ is necessarily determined via $L^\star _T(\gamma ) =\delta$. As we observe a jump discontinuity of $\gamma \mapsto L^\star _T(\gamma )$, this suggests that there is a duality gap at least for certain values of $\delta$.

We proceed by comparing the costs and losses under the optimal strategy with some other heuristic strategies. As first benchmark, we consider a uniform strategy by which the central agent injects cash at a constant rate $b_{\max }$ whenever an agent’s value $X_t\le c$ for a constant *c*, which we vary, resulting in pairs $(C_T^{\textrm{u}}(c), L_T^{\textrm{u}}(c))$. The total cost here can be computed as $C_T^{\textrm{u}}(c) = b_{\max } \cdot \int _0^T \int _0^c p^{\textrm{u}}(t,x) \, dx dt$, where $p^{\textrm{u}}$ is the density of the regularised process with such uniform (in time) control.

We also consider a ‘front loaded’ strategy whereby at the outset, for some chosen ‘floor’ $d>0$, the central agent injects a lump sum of $d-X_{0-}$ into all players with $X_{0-}<d$, hence lifting their reserves up to *d*. Again, we vary *d* to obtain a parametrised curve $(C_T^{\textrm{f}}(d), L_T^{\textrm{f}}(d))$. The total cost in this case is found as $C_T^{\textrm{f}}(d) = \int _0^d (d-x) f(x) \, dx$.

**Fig. 8:**
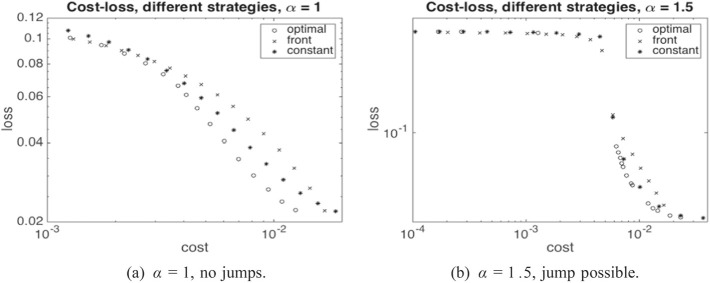
Cost-loss pairs $(C^\star _T, L^\star _T)$ under optimal strategy compared to those for a constant strategy, $(C_T^{\textrm{u}}, L_T^{\textrm{u}})$, and front-up strategy, $(C_T^{\textrm{f}}, L_T^{\textrm{f}})$, for two values of $\alpha$

**Fig. 10:**
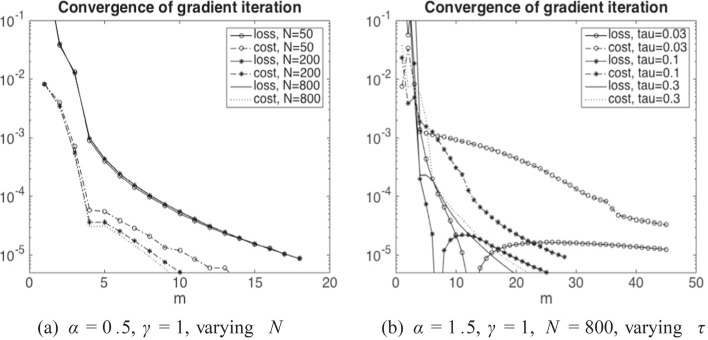
Convergence of *C* and *L* over policy gradient iterations

The pairs of cost and loss are shown in Fig. 8. In particular, Fig. 8a illustrates the case without jump for $\alpha =1$, whereas in the situation of 8b with $\alpha =1.5$ there is a jump in the uncontrolled system, which can be avoided with sufficiently large control.

In both cases, the optimal strategy gives lower losses than the heuristic strategies for the same fixed cost. Conversely, less cash injection is required for a given loss tolerance.^5^

We observe that we cannot enforce the *sufficient* condition for avoiding jumps, i.e., $\alpha \Vert f\Vert _{\infty } < 1$, for any of these strategies. It is clear that the strategy where the initial capital of all banks is raised to a certain minimum level *d* satisfies $\mathbb {E}[X_{0-}]\ge d$ and hence the *necessary* condition for avoiding jumps, $\mathbb {E}[X_{0-}] \ge \alpha /2$, holds for $d \ge \alpha /2$. However, the sufficient condition can be violated even when all banks have a high initial capital. What would work to enforce the sufficient condition is to set $X_{0-} \sim U(d, d+\alpha +\varepsilon )$ for some $\varepsilon >0$, $d \ge 0$ and *U* the uniform distribution on $[d,d+\alpha +\varepsilon ]$.

Considering the physical jump condition (4), for a jump to occur it matters how much of the surviving mass can be concentrated around zero at any given point. Intuitively, starting with a higher initial condition, the Brownian motion will diffuse the mass sufficiently and make large concentrations at zero less likely, hence preventing a jump. Similarly, sufficiently large $\beta (t,x)$ for small *t* and *x* should transport mass away from zero and prevent a jump as long as the initial density satisfies $f(0+)<1/\alpha$ (which rules out an instantaneous jump). Therefore, both the constant and optimal strategies should be able to prevent jumps for large enough $b_{\max }$. A rigorous analysis, however, goes beyond the scope of this paper.

## Figures and Tables

**Fig. 9:**
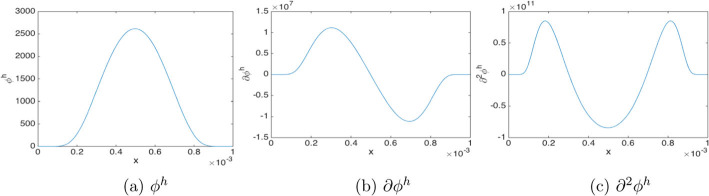
Smoothed Dirac delta and its derivatives, for $h=10^{-3}$

## Proofs

### Notation

Throughout the paper, $D([-1,\infty ))$ denotes the space of càdlàg functions on $[-1,\infty )$ endowed with the $M_1$-topology, $C([0,\infty ))$ denotes the space of continuous functions on $[0,\infty )$ endowed with the topology of compact convergence, i.e., $f_n \rightarrow f$ in $C([0,\infty ))$ if and only if $f_{n}\vert _{K} \rightarrow f\vert _{K}$ uniformly for every compact $K \subseteq [0,\infty )$. If *S* is a Polish space, we denote the space of probability measures on *S* by $\mathcal {P}(S)$ and endow it with the topology of weak convergence, i.e., we say that $\mu _n \rightarrow \mu$ in $\mathcal {P}(S)$ iff $\int _{S} F(x) \textrm{d}\mu _n (x) \rightarrow \int _{S}F(x) \textrm{d}\mu (x)$ for all $F \in C_b (S;\mathbb {R})$. If $\mu \in \mathcal {P}(S)$ and $F :S \rightarrow \mathbb {R}$, we denote the integral of *F* with respect to $\mu$ also with brackets, i.e., we write $\int _{S}F(x)~\textrm{d}\mu (x) = \langle \mu , F \rangle$. Furthermore, if $\nu$ is the pushforward of the measure $\mu$ with respect to the map *T*, we denote this by $T(\mu ) = \nu$.

### Existence of minimal solutions and optimizers

#### Lemma A.1

For any $\beta \in \mathcal {B}_T$, define the process

57 $$\begin{aligned} Z_t = X_{0-}+\int _{0}^{t}\beta _s\,ds + B_t, \quad t \ge 0. \end{aligned}$$

Then, the process *Z* satisfies the extended crossing property, i.e.,

58 $$\begin{aligned} \mathbb {P}\left( \inf _{0\le s\le h} (Z_{\tau +s} - Z_{\tau }) = 0 \right) = 0, \quad h > 0, \end{aligned}$$

for any stopping time $\tau$ with respect to $(\mathcal {F}_{t})_{t \ge 0}$, the natural filtration generated by *Z*.

#### Proof

Let $\tau$ be a $(\mathcal {F}_t)_{t\ge 0}-$stopping time. Since $\beta \in \mathcal {B}_T$ is almost surely bounded, by Novikov’s condition and Girsanov’s theorem we may find an equivalent probability measure $\mathbb {Q}$ such that *Z* is a $(\mathcal {F}_t)_{t \ge 0}$-Brownian motion under $\mathbb {Q}$. The strong Markov property of Brownian motion then yields

$$\begin{aligned} \mathbb {Q}\left( \inf _{0\le s\le h} (Z_{\tau +s} - Z_{\tau }) = 0 \right) = \mathbb {Q}\left( \inf _{0\le s\le h} Z_s = 0 \right) = 0, \quad h > 0 \end{aligned}$$

and by the equivalence of $\mathbb {P}$ and $\mathbb {Q}$ the claim follows. $\square$

### Existence of solutions

#### Lemma A.2

Suppose that $f_n \rightarrow f$ in $S_T$. Then, $\int ^{\cdot } f_n(s)\,ds \rightarrow \int ^{\cdot } f(s)\,ds$ uniformly in *t* on any compact subset of $[0,\infty )$.

#### Proof

Let $T' > 0$. By weak $L^2([0,\infty ))$-convergence, we have $\int _{0}^{t} f_n(s)\,ds \rightarrow \int _{0}^{t} f(s)\,ds$ for any $t \in [0,T']$. Let $\epsilon > 0$ and choose $t_k$ with $0 = t_0 \le t_1 \le \dots \le t_m = T'$ such that

59 $$\begin{aligned} \int _{t_i}^{t_{i+1}} f(s)\,ds < \epsilon /2, \quad i \in \{0,\dots ,m-1\}. \end{aligned}$$

Choose *n* large enough such that $\left| \int _{0}^{t_i} f_n(s)-f(s)\,ds\right| < \epsilon /2$ for all $i \in \{0,\dots ,m\}$. We obtain, for $t \in [t_i,t_{i+1}]$,

$$\begin{aligned}&\int _{0}^{t} f_n(s)\,ds - \int _{0}^{t} f(s)\,ds \le \int _{0}^{t_{i+1}} f_n(s)\,ds - \int _{0}^{t_{i+1}} f(s)\,ds + \epsilon /2 \le \epsilon , \\&\int _{0}^{t} f_n(s)\,ds - \int _{0}^{t} f(s)\,ds \ge \int _{0}^{t_{i}} f_n(s)\,ds - \int _{0}^{t_{i}} f(s)\,ds - \epsilon /2 \ge -\epsilon , \end{aligned}$$

which yields the claim. $\square$

#### Proof of Theorem 2.2

Step 1: We construct the reference probability space ${\mathscr {S}}$.

Since the sequence $\operatorname {law}((X_{0-}^n,B^n))$ is constant and the space $[0,\infty ) \times C([0,\infty ))$ (endowed with the product topology of Euclidean and uniform topology) is Polish, the sequence $(X_{0-}^n,B^n)$ is tight on $[0,\infty ) \times C([0,\infty ))$. Since $S_T$ is compact, the sequence $(X_{0-}^n,B^n,\beta ^n)$ is tight on $[0,\infty ) \times C([0,\infty ))\times S_T$. By Prokhorov’s theorem, after passing to a subsequence if necessary, we may assume that $\operatorname {law}((X_{0-}^n,B^n,\beta ^n)) \rightarrow \operatorname {law}((X_{0-},B,\beta ))$ in $\mathcal {P}([0,\infty ) \times C([0,\infty )) \times S_T)$. By the Skorokhod representation theorem, we may without loss of generality assume that $(X_{0-}^n,B^n,\beta ^n) \rightarrow (X_{0-},B,\beta )$ holds almost surely on some probability space, which we denote by ${\mathscr {S}} = (\Omega , \mathcal {F}, \mathbb {P})$. Note that this is possible since the property of $\Lambda$ solving (7)–(8) only depends on the (joint) law of $(X_{0-},B,\beta )$. We then define $\mathcal {F}_t$ to be $\sigma (\{(X_{0-},B_s,\int _{0}^{s} \beta _u \,ds), s \le t \})$ for $t \ge 0$. Since $\beta$ is measurable and $(\mathcal {F}_t)_{t \ge 0}$-adapted, it admits a progressively measurable modification, which we again denote by $\beta$, and we see that $\beta \in \mathcal {B}_T({\mathscr {S}})$. We next show that *B* is a $(\mathcal {F}_t)_{t \ge 0}$-Brownian motion: by the continuous mapping theorem and the continuity of the coordinate projections, it follows that $\operatorname {law}(B)$ is the Wiener measure. Let $0< r_1< \dots< r_k< s < t$ and let $f_i \in C_b(\mathbb {R}^2;\mathbb {R}), i=1,\dots ,k$ and $g \in C_b(\mathbb {R};\mathbb {R})$ and set $F(w^1,w^2):= \prod _{i=1}^{k} f_i (w_{r_i}^1,w_{r_i}^2)$, then we have by dominated convergence

$$\begin{aligned} \mathbb {E} \left[ F\left( B,\int _{0}^\cdot \beta _u \,du\right) g(B_t - B_s) \right]&= \lim _{n\rightarrow \infty }\mathbb {E} \left[ F\left( B^n,\int _{0}^\cdot \beta _u^n\,du\right) g(B_t^n - B_s^n) \right] \\&= \lim _{n\rightarrow \infty }\mathbb {E} \left[ F\left( B^n,\int _{0}^\cdot \beta _u^n\,du\right) \right] \mathbb {E} \left[ g(B_t^n - B_s^n) \right] \\&= \mathbb {E}\left[ F\left( B,\int _{0}^\cdot \beta _u\,du\right) \right] \mathbb {E} \left[ g(B_t - B_s) \right] . \end{aligned}$$

Since the Borel $\sigma$-algebra on $C([0,\infty ))$ is generated by the evaluation mappings, we obtain that $B_t - B_s$ is independent of $\mathcal {F}_s$ for $0< s < t$, and therefore *B* is an $(\mathcal {F}_t)_{t \ge 0}$-Brownian motion. We have shown that ${\mathscr {S}}$ is an admissible reference space.

Step 2: Since *M* is compact, after passing to subsequences if necessary, we may assume that $\frac{1}{\alpha } \Lambda ^n \rightarrow \frac{1}{\alpha }\Lambda$ in *M*. We now show that $(X_{0-},B,\beta ,\Lambda )$ solves (7)–(8) on ${\mathscr {S}}$. Let $\bar{E}= C([0,\infty )) \times M$ be endowed with the product topology and define $Z_t^n:=X_{0-}^n + B_t^n + \int _{0}^{t} \beta _s^{n}\,ds$ and *Z* analogously as $Z_t = X_{0-} + B_t + \int _{0}^{t} \beta _s\,ds$. By Lemma A.2, it follows that $Z^n\rightarrow Z$ in $C([0,\infty ))$ almost surely. Since $\Lambda$ is deterministic, it follows that $\xi ^n:= (Z^n,\frac{1}{\alpha }\Lambda ^n) \rightarrow (Z,\frac{1}{\alpha }\Lambda ) =: \xi$ in distribution on $\bar{E}$. We introduce some notation: define $\iota :\bar{E}\rightarrow D([-1,\infty ))$ for $w \in C([0,\infty ))$ and $\ell \in M$ as

60 $$\begin{aligned} \iota (w,\ell )_t := {\left\{ \begin{array}{ll} w_0, \quad & t \in [-1,0), \\ w_t - \alpha \ell _t, \quad & t \in [0,\infty ). \end{array}\right. } \end{aligned}$$

For $t \in \mathbb {R}$ and $x\in D([-1,\infty ))$, define the path functionals $\tau _0 (x):= \inf \{s \ge 0: x_s \le 0\}$ and $\lambda _t (x):= \mathbb {1}_{\{\tau _0 (x) \le t\}}$. Then, $(X_{0-},B,\beta ,\Lambda )$ is a solution to (7)–(8) on ${\mathscr {S}}$ if and only if $\alpha \mathbb {P}(\tau _0 (Z - \Lambda ) \le t) = \Lambda _t$. We may write this condition equivalently on the canonical path space $D([-1,\infty ))$ as $\alpha \langle \iota (\xi ),\lambda _t \rangle = \Lambda _t$ for $t \ge 0$. Note that with the notational conventions explained in Sect. A.1, $\iota (\xi )$ denotes the pushforward of the measure $\xi$ by the map $\iota$ and $\langle \iota (\xi ),\lambda _t \rangle$ denotes the integral of the functional $\lambda _t$ with respect to $\iota (\xi )$. Since *Z* satisfies the extended crossing property by Lemma A.1, $\iota (\xi )$ satisfies the crossing property (cf the proof of Lemma 5.5 in Cuchiero et al. (2023)). Lemma 5.3 in Cuchiero et al. (2023) and Step 1 imply that

61 $$\begin{aligned} \Lambda = \lim _{n\rightarrow \infty }\Lambda ^n = \lim _{n\rightarrow \infty } \alpha \langle \iota (\xi ^n),\lambda \rangle = \alpha \langle \iota (\xi ),\lambda \rangle . \end{aligned}$$

in *M*. $$\square $$

### Proof of Theorem 2.7

#### Proof

The proof is to a large extent analogous to the proof of Proposition 5.6 in Cuchiero et al. (2023). Define

$$\begin{aligned} \xi _N:= \frac{1}{N} \sum _{i=1}^{N} \delta _{(X_{0-}^{i,N} + B^{i,N},\beta ^{i,N},L^N)}. \end{aligned}$$

Since $\xi _N$ is a random probability measure on $C([0,\infty )) \times S_T \times M$ and the spaces $S_T$ and *M* are compact, $\xi _N$ is tight by the same reasoning as in Corollary 4.5 in Cuchiero et al. (2023). Therefore, after passing to a subsequence if necessary, we may assume that $\operatorname {law}(\xi _N) \rightarrow \operatorname {law}(\xi )$ for some random probability measure $\xi$ on $C([0,\infty )) \times S_T \times M.$ By Skorokhod representation, we may assume without loss of generality that the convergence happens almost surely on the same probability space ${\mathscr {S}}$. Arguing in the same fashion as in the proof of Lemma 5.4 in Cuchiero et al. (2023), we see that for almost every $\omega \in \Omega$, if $\operatorname {law}((W,\beta ,{\textsf{L}})) = \xi (\omega )$, then $W-W_{0}$ is a Brownian motion with respect to the filtration generated by $(W,\int _{0}^{\cdot }\beta _s\,ds,{\textsf{L}})$. For $(w,b,\ell ) \in C([0,\infty )) \times S_T \times M$, set $\hat{\iota }(w,b,\ell )_t:= \iota (w,\ell )_t + \int _{0}^{t} b_s\,ds$, where $\iota$ is defined as in (60). By Lemma A.2, the map $b \mapsto \int _{0}^{\cdot } b_s\,ds$ is continuous from $S_T$ to $C([0,\infty ))$, and therefore also as a map from $S_T$ to $D([-1,\infty ))$. Theorem 4.2 in Cuchiero et al. (2023) together with Corollary 12.7.4 in Whitt (yyy) then shows that $\hat{\iota }$ is continuous. Since $\hat{\iota }(\xi _N) = \mu _N$, the continuous mapping theorem implies that $\hat{\iota }(\xi ) = \mu$. Applying the continuous mapping theorem to $(w,b,\ell ) \mapsto w_0$, we see that $\operatorname {law}(W_0) = \nu _0$ holds almost surely. It remains to check that if $\operatorname {law}(W,\beta ,{\textsf{L}}) = \xi (\omega )$, then ${\textsf{L}}_t \equiv \langle \mu (\omega ),\lambda _t \rangle$ for $t \ge 0$ holds $\xi (\omega )$-almost surely for almost every $\omega \in \Omega$. This can be checked as in Step 1 of the proof of Proposition 5.6 in Cuchiero et al. (2023), making use of Lemma A.1 to show that $\mu (\omega )$ satisfies the crossing property (almost surely). $\square$

## Further numerical details and tests

We here report further details and tests of our numerical procedure.

### Smoothing

Let us start by precisely specifying the smoothing functions $\phi ^h$ and $\Phi ^h$ used in the regularization procedure of the objective function and the dynamics. We choose $\phi ^h(x) = \phi (x)/h$, where,

62 $$\begin{aligned} \phi (x) = {\left\{ \begin{array}{ll}\frac{1}{\mathcal {I}}\exp ( - 1/(x(1 - x)), & x \in [0,1],\\ 0 & \hbox {else}. \end{array}\right. } \end{aligned}$$

where $\mathcal {I} = 0.007029858406609$ normalises the integral (close) to 1. The function $\phi ^h$ and its first two derivatives are shown in Fig. 9 for $h=10^{-3}$. Note in particular the large positive and negative values of $\partial ^2 \phi ^h$.

**Table 2:** Mesh convergence, 100 $\cdot$ costs, $\alpha =1.5$ and $\gamma =0.1$

			$$10^3 \cdot h$$	1	$$2^{-1}$$	$$2^{-2} $$	$$2^{-3}$$	$$2^{-4} $$	$$2^{-5} $$
$\frac{N}{10^2}$	$N_x/10^3$	$10^3 \cdot \theta _N$	$\rho _N$						
1	3.75	$-$1.650	1.86	1.2548	1.1449	1.0225	0.8010	0.0526	0.5638
2	7.5	$-$0.887	0.83	1.2383	1.1115	1.0085	0.9004	0.6965	0.0343
4	15	$-$1.066	1.99	1.2294	1.0970	0.9939	0.9246	0.6131	0.4506
8	30	$-$0.534	2.00	1.2187	1.0846	0.9839	0.9126	0.8484	1.0968
16	60	$-$0.267	–	1.2134	1.0784	0.9790	0.9126	0.8644	0.8199
32	120	–	–	1.2107	1.0753	0.9764	0.9123	0.8701	0.8365
			$10^3 \cdot \vartheta _h$	$-$1.354	$-$0.989	$-$0.641	$-$0.422	$-$0.335	–
			$\varrho _h$	1.37	1.54	1.51	1.25	–	–

We also choose for some $\kappa >0$, $\Phi ^h(x) = \Phi (x/(\kappa h))$, where $\Phi (x) = \mathcal {N}((x+1/2)/(x*(1+x)))$, $x\in (-1,0)$, and 0 else, with $\mathcal {N}$ the standard normal CDF.

### Mesh convergence

Here, we demonstrate the convergence of the finite difference approximation in two more settings. In Table 2 for the cost, for $\alpha = 1.5$, and in Table 2 again for the losses, but now for $\alpha = 0.5$. The notation with $\theta _N$ $\rho _N$, $\vartheta _N$ and $\varrho _h$ is analogous as before.

**Table 3:** Mesh convergence, 100 $\cdot$ losses, $\alpha =0.5$ and $\gamma =0.1$

			$$10^3 \cdot h$$	1	$$2^{-1}$$	$$2^{-2} $$	$$2^{-3}$$	$$2^{-4} $$	$$2^{-5} $$
$\frac{N}{10^2}$	$N_x/10^3$	$10^3 \cdot \theta _N$	$\rho _N$						
1	3.75	3.802	1.99	7.3549	7.4198	7.6336	8.7134	735.42	0
2	7.5	1.910	2.03	7.3929	7.4573	7.4919	7.6925	8.7711	735.91
4	15	0.941	2.00	7.4120	7.4771	7.5103	7.4765	9.5999	$-$4.196
8	30	0.470	2.00	7.4214	7.4868	7.5203	7.5373	7.5867	6.2291
16	60	0.235	–	7.4261	7.4916	7.5251	7.5421	7.5504	7.5550
32	120	–	–	7.4285	7.4939	7.5275	7.5445	7.5531	7.5571
			$10^3 \cdot \vartheta _h$	0.6547	0.3353	0.1701	0.0860	0.0398	–
			$\varrho _h$	1.95	1.97	1.97	2.15	–	–

In these settings, the behaviour in *h* is somewhat better than in Table 1 (losses for $\alpha = 1.5$, i.e. with jump in the uncontrolled case), but as there, a good approximation is only achieved if the mesh size is sufficiently small in comparison with *h*.

### Gradient iteration

Finally we conducted further tests in view of the mesh refinement and the role of the step size $\tau$ in the gradient iteration.

Figure 10a, left, illustrates that the convergence is robust with respect to mesh refinement, i.e., the number of iterations required for a prescribed accuracy does not increase significantly as the number of time steps and mesh points increases simultaneously.

In Fig. 10b we investigate the effect of the step size $\tau$. Choosing $\tau$ small leads to poor convergence, while $\tau =0.3$ is optimal among the values presented here. Picking even larger step sizes can lead to divergence.

## Footnotes

1 As we are working in continuous time, we also assume that the central agent is able to inject money continuously. At first sight this is a bold approximation of reality but allows us to work without an a-priori fixed time grid determining when the central agent reacts.

2 We consider $L_{T-}$ rather than $L_{T}$ in the constraint because $\Lambda$ may have a jump discontinuity precisely at *T*, which would considerably complicate the analysis. However, by Ledger and Søjmark (2021, Corollary 2.3), we know that solutions to (1) cannot have discontinuities after time $\alpha ^2/(2\pi )$. We can derive an analogous result for the controlled case using Girsanov’s theorem if, in addition to the pointwise bound on $\beta$, we assume a bound on the total cost over the infinite horizon, i.e. $\int _{0}^{\infty } \beta _s~\textrm{d}s \le c_{\text {max}}$ for some $c_{\text {max}} > 0$. In this setting, by choosing *T* sufficiently large, we then do not need to distinguish between $L_{T-}(\beta )$ and $L_{T}(\beta )$.

3 Proving the absence a duality gap seems difficult as standard minimax theorems cannot be easily applied. Moreover, our numerical experiments suggest that at least for certain values of $\delta$ it might fail to hold true.

4 Computations performed using Matlab on a 2.8 GHz Intel Core i7 with 16 GB 1600 MHz DDR3.

5 Note that the strategy with upfront payments is not in the class of Lipschitz feedback controls for which the policy gradient method is designed.
